# Effects of tannic acid supplementation on growth performance, gut health, and meat production and quality of broiler chickens raised in floor pens for 42 days

**DOI:** 10.3389/fphys.2022.1082009

**Published:** 2022-12-16

**Authors:** Janghan Choi, Guanchen Liu, Doyun Goo, Jinquan Wang, Brain Bowker, Hong Zhuang, Woo Kyun Kim

**Affiliations:** ^1^ Department of Poultry Science, University of Georgia, Athens, GA, United States; ^2^ US National Poultry Research Center, USDA-ARS, Athens, GA, United States

**Keywords:** tannic acid, gut microbiota, gut health, floor pen, pelleting process, meat production, and fat accumulation

## Abstract

A study was conducted to investigate the effects of tannic acid (TA) supplementation on growth performance, gut health, antioxidant capacity, gut microbiota, and meat yield and quality in broilers raised for 42 days. A total of 700 one-day-old male broiler chickens (Cobb500) were allocated into 5 treatments with 7 replicates of 20 birds per pen. There were five treatments: 1) tannic acid 0 (TA0: basal diet without TA); 2) tannic acid 0.25 (TA0.25: basal diet+0.25 g/kg TA); 3) tannic acid 0.5 (TA0.5: basal diet+0.5 g/kg TA); 4) tannic acid 1 (TA1: basal diet+1 g/kg TA); and 5) tannic acid 2 (TA2: basal diet+2 g/kg TA). The dietary phases included starter (D 0 to 18; crumble feed), grower (D 18 to 28; pellet feed), and finisher (D 28 to 42; pellet feed). On D 18, the supplementation of TA linearly reduced body weight (BW) and average daily feed intake (ADFI) (*p* < 0.05), and on D 28, the supplementation of TA linearly reduced BW, average daily gain (ADG), and feed conversion ratio (FCR) (*p* < 0.05). Relative mRNA expression of genes related to mucin production (*MUC2*), tight junction proteins (*CLDN2* and *JAM2*), and nutrient transporters (*B0AT1* and *SGLT1*) was linearly increased by the supplementation of TA (*p* < 0.05). The supplementation of TA tended to linearly increase the relative abundance of the family Enterobacteriaceae (*p* = 0.08) and quadratically increased the relative abundance of the families Lachnospiraceae and Ruminococcaceae in the cecal microbial communities (*p* < 0.05). On D 36, the ratio of the phyla Firmicutes and Bacteroidetes was quadratically reduced by the supplementation of TA (*p* < 0.05). On D 42, bone mineral density and the lean to fat ratio were linearly decreased by the supplementation of TA (*p* < 0.05). On D 43, total chilled carcass weight was linearly reduced (*p* < 0.05), and proportion of leg weight was increased by supplementation of TA (*p* < 0.05). The supplementation of TA linearly reduced pH of the breast meat (*p* < 0.05) and linearly increased redness (a*) (*p* < 0.05). Although the supplementation of TA positively influenced gut health and gut microbiota in the starter/grower phases, it negatively affected overall growth performance, bone health, and meat production in broilers on D 42.

## 1 Introduction

In the past, antibiotic growth promoters (AGP) have been supplemented to broiler diets to enhance growth performance and gut health and to prevent diseases in broilers (Caly et al., 2015). Due to the public concerns about the spread of antibiotic resistant bacteria and their genes, there is a global movement to implement antibiotic-free production in the poultry industry (Haque et al., 2020). However, withdrawal of AGP without appropriate alternative strategies against bacterial infections could result in reduced production efficiency and broiler health and welfare issues by inducing severe microbial infection in broilers (Cervantes, 2015). It has been essential for the poultry industry to find appropriate bioactive compounds that can improve growth performance and gut health in antibiotic-free production. Diverse bioactive compounds, such as essential oils (Yang et al., 2021), amino acids (Teng et al., 2021), organic acids (Adil et al., 2010), plant extracts (Mogire et al., 2021; Yadav et al., 2022), and exogenous enzymes (Lu et al., 2020), have been studied and used as AGP alternatives in poultry production. Alternatives for AGP should be able to enhance growth performance, gut health, and meat production and quality of broilers and should be safe to the public and eco-friendly and cost-effective in broiler production (Yang et al., 2015).

Tannins, polyphenol compounds that can precipitate proteins, are considered as AGP alternatives in broiler production due to their effective antimicrobial, antioxidative, and anti-inflammatory effects in chickens (Choi and Kim, 2020). Tannic acid (TA), which is composed of 7–8 gallic acids molecules and one glucose molecule as a central core, is a standard of hydrolysable tannins and present in woods such as oak, chestnut, and acacia (Romani et al., 2006). Traditionally, TA was considered as an anti-nutritional factor due to its protein precipitation capacity, which can result in reduced nutrient digestibility and proteolytic activity in the liver of chickens (Marzo et al., 2002; Redondo et al., 2014). Many recent studies showed that the supplementation of TA at appropriate dosages (up to 2 g/kg) improved growth performance, gut health, immune system, and gut microbiota in broilers under non-challenge conditions and diverse challenging conditions (*Eimeria* spp., *Salmonella* spp., etc.) (Diaz Carrasco et al., 2018; Tonda et al., 2018; Ramah et al., 2020). Our previous study (Choi et al., 2022d) demonstrated that 0.5 g/kg TA increased activities of endogenous antioxidant enzymes, whereas higher than 1 g/kg TA exhibited antinutritional effects in broilers on D 21. However, it is still uncertain whether the supplementation of TA (up to 2 g/kg) would beneficially or negatively influence growth performance, gut health, antioxidant capacity, gut microbiota, and meat production and quality in broilers on D 42 (slaughter age). Therefore, this study was aimed to investigate the effects of TA supplementation (up to 2 g/kg TA) on growth performance, gut health, antioxidant capacity, gut microbiota, and meat yield and quality in broilers on D 42.

## 2 Materials and methods

### 2.1 Animals, diets, experimental design, and growth performance

The current study was reviewed and approved by the Institutional Animal Care and Use Committee at the University of Georgia, Athens, GA. A total of 700 one-day-old Cobb 500 male broiler chickens were randomly allotted to 5 treatments with 7 replicates of 20 birds per pen in a completely randomized design. The five treatments included 1) tannic acid 0 (TA0; basal diet without TA); 2) tannic acid 0.25 (TA0.25; basal diet + 0.25 g/kg TA); 3) tannic acid 0.5 (TA0.5; basal diet + 0.5 g/kg TA); 4) tannic acid 1 (TA1; basal diet + 1 g/kg TA); and 5) tannic acid 2 (TA2; basal diet + 2 g/kg TA). The TA (> 99% purity; Chinese natural gall nuts) was purchased from Sigma-Aldrich Co. (St Louis, MO) and was included in the entire experimental period. Before TA was added to the basal diets, TA was premixed with 10 kg basal diets. The experiment period was divided into starter (D 0 to 18; crumble feed), grower (D 18 to D 28; pellet feed), and finisher (D 28 to 42; pellet feed) phases, and the diets were formulated to meet or exceed recommendation levels according to the Cobb 500 nutrient requirement guide (2018) (Table 1). Conditioning temperature was 80°C for the feed pelleting process. On D 28 to 35, all diets included 0.3% titanium dioxide (Acros Organics, Morris Plains, NJ) as an inert marker to determine nutrient digestibility. Birds were raised in floor pens (width: 1.52 m, length: 1.22 m, height: 0.61 m) equipped with one feeder and three drinker nipples per pen, and birds had free access to water and feed. Temperature and light were controlled in accordance with the recommendation of the Cobb 500 broiler management guide (2018). Body weight (BW) and feed disappearance were measured on D 18, 28, and 42 to calculate average daily gain (ADG), average daily feed intake (ADFI), and feed conversion ratio (FCR).

**TABLE 1:** Ingredients and nutrient compositions of basal diets (As-fed basis).

Items	D 0 to 18	D 18 to 28	D 28 to 42
Feed form	Crumble	Pellet	Pellet
Ingredients (kg/ton)			
Corn	614.01	672.67	690.87
Soybean meal (480 g crude protein/kg)	323.935	263.16	239.22
Dicalcium phosphate	15.164	12.622	12.82
Filler^a^	10	10	10
Soybean oil	12.433	18.58	24
limestone	11.564	10.571	10.655
dl-Methionine 99%	2.912	2.656	2.486
l-Lysine HCl 78%	1.974	2.003	2.144
Vitamin Premix^b^	2.5	2.5	2.5
Common Salt	3.417	3.455	3.47
l-threonine	0.791	0.475	0.538
Mineral Premix^c^	0.8	0.8	0.8
Coccidiostats^d^	0.5	0.5	0.5
Total	1,000	1,000	1,000
Calculated energy and nutrient value, %			
Metabolizable energy, kcal/kg	3,000	3,100	3,150
Crude protein	20.5	18	17
SID^d^ Methionine	0.598	0.54	0.51
SID^d^ Total sulfur amino acids	0.88	0.8	0.76
SID^d^ Lysine	1.17	1.02	0.97
SID^d^ Threonine	0.78	0.66	0.63
Total calcium	0.87	0.76	0.76
Available phosphate	0.435	0.38	0.38

^a^ Sand and tannic acid were included to obtain wanted tannic acid dosages in the feed as follows: Tannic acid 0 (TA0): sand 10 g/kg + tannic acid 0 g/kg; Tannic acid 0.25 (TA0.25): sand 9.75 g/kg + tannic acid 0.25 g/kg; Tannic acid 0.5 (TA0.5): sand 9.5 g/kg + tannic acid 0.5 g/kg; Tannic acid 1 (TA1): sand 9 g/kg + tannic acid 1 g/kg; and Tannic acid 2 (TA2): sand 8 g/kg + tannic acid 2 g/kg. Tannic acid was purchased from Sigma–Aldrich (St. Louis, MO). In the finisher phase, titanium dioxide 3 g/kg (Acros Organics, Morris Plains, NJ) was included in the sand part.

^b^ Vitamin mix provided the following in mg/100 g diet: thiamine-HCl, 1.5; riboflavin 1.5; nicotinic acid amide 15; folic acid 7.5; pyridoxine-HCl, 1.2; d-biotin 3; vitamin B-12 (source concentration, 0.1%) 2; d-calcium pantothenate 4; menadione sodium bisulfite, 1.98; α-tocopherol acetate (source 500,000 IU/g), 22.8; cholecalciferol (source 5000,000 IU/g) 0.09; retinyl palmitate (source 500,000 IU/g), 2.8; ethoxyquin, 13.34; I-inositol, 2.5; dextrose, 762.2.

^c^ Mineral mix provided the following in g/100 g diet: Ca (H_2_PO_4_)_2_ · H_2_O, 3.62; CaCO_3_, 1.48; KH_2_PO_4_, 1.00; Na_2_SeO_4_, 0.0002; MnSO_4_ · H_2_O, 0.035; FeSO_4_ · 7H_2_O, 0.05; MgSO_4_ · 7H_2_O, 0.62; KIO_3_, 0.001; NaCl, 0.60; CuSO_4_ · 5H_2_O, 0.008; ZnCO_3_, 0.015; CoCl_2_ · 6H_2_O, 0.00032; NaMoO_4_ · 2H_2_O, 0.0011; KCl, 0.10; dextrose, 0.40.

^d^ Coban 90, Elanco Animal Health, Greenfield, IN.

^e^ SID: standard ileal digestible amino acid.

### 2.2 Sampling, dual-energy X-ray absorptiometry, litter ammonia, and foot pad lesion

On D 18 and 36, one bird per pen was randomly selected and euthanized *via* cervical dislocation to collect samples of liver, intestinal tissue (mid-duodenum, mid-jejunum, and mid-ileum), and cecal content. All tissue samples were washed with PBS to remove remaining digesta and blood. Samples of liver, mid-jejunum tissue, and cecal content were snap-frozen and stored at –80°C for further analyses. For intestinal morphology, mid-duodenum, mid-jejunum, and mid-ileum samples were fixed in a 10% formaldehyde solution. On D 36, randomly selected four birds were euthanized, and digesta samples from 10 cm below Meckel’s diverticulum to the upper 10 cm of the ileo-cecal-colic junction were collected and oven-dried at 75°C until constant weight was achieved. On D 42, randomly selected one bird per pen was euthanized *via* cervical dislocation and scanned using dual-energy X-ray absorptiometry (DEXA, GE Healthcare, Madison, WI) to determine total tissue weight (g), bone mineral content (BMC; g), bone mineral density (BMD; g/cm^2^), lean weight (g), fat weight (g), body fat percentage (%), and lean:fat (g/g). On D 42, severity of foot pad dermatitis (FPD) was measured from all birds in each pen according to Eichner et al. (2007): score 0: no lesion; score 1: FPD covers less than 25% of the food pad; score 2: FPD covers 25%–50% of the food pad; and score 3: FPD covers more than 50% of the food pad. Both foot pads were checked in birds, and scores from both foot pads were averaged, and FPD incidence (%) was also calculated. Ammonia level (mg/kg) on the litter was measured using a Chillgard^®^ RT Refrigerant Monitor (MSA, Cranberry Township, PA) connected to a HOBO^®^ monitoring station (Onset, Bourne, MA) according to Aston et al. (2019).

### 2.3 Apparent ileal digestibility of dry matter, organic matter, ash, crude protein, and crude fat

Oven-dried feed (75°C till constant weight; 0.5 g) and ileal digesta (0.3 g) samples were ashed at 600°C overnight, and concentrations of titanium dioxide were determined according to Short et al. (1996). The concentration of crude protein (CP) was analyzed using nitrogen combustion analyses according to AOAC international (2000) analytical method 990.03. The crude fat (CF) was determined according to AOAC international (2000) analytical method 942.05. Apparent ileal digestibility (AID) of dry matter (DM), organic matter (OM), ash, CP, and CF was calculated according to Lin and Olukosi (2021).

### 2.4 Intestinal morphology

After 72 h of fixation in 10% formalin solution, the fixed intestine samples were embedded in paraffin and cut into 4 μm, and the samples were stained with hematoxylin and eosin (H&E). The images of H&E-stained slides were taken using a microscope (BZ-X810; Keyence, Osaka, Japan). Five well-shaped villus and their corresponding crypts were selected per slide, and villus height (VH) and crypt depth (CD) were measured by using ImageJ (National Institutes of Health, Bethesda, MD). The VH to CD ratio (VH:CD) was calculated for each villi and crypt.

### 2.5 Jejunal brush border digestive enzyme activities and serum alkaline phosphatase

Around 100 mg of mid-jejunum (whole tissue) samples were homogenized in 1.8 ml PBS using a bead beater (Biospec Products, Bartlesville, OK). Afterwards, the samples were centrifuged at 12,000 × *g* for 15 min at 4°C, and the protein concentrations of the supernatants was determined using Pierce BCA protein assay kits according to the manufacturer’s instructions (Thermo Fisher Scientific, Waltham, MA). Activities of maltase and sucrase in the supernatants were analyzed according to the method of Lackeyram (2012). Briefly, 100 μl supernatants were mixed with 400 μl maltose (75 mM) and sucrose solution (75 mM), separately and incubated at 41°C for 30 min. Afterwards, the concentrations of glucose were determined using a Glucose Oxidase Reagent Set (Pointe Scientific, Canton, MI) according to the manufacturer’s protocol. To determine activities of lipase in the supernatants, the 10 times diluted supernatants (60 μl) were incubated with 1 mg/ml *p*-nitrophenyl palmitate solution (Sigma-Aldrich Co., St Louis, MO; 140 μl) at 41°C for 30 min according to the method of Elgharbawy et al. (2018). The activities of leucine aminopeptidase (LAP) were assayed by incubating 100 μl supernatant with 100 μl 1 mg/ml l-leucine-*p*-nitroanilide solution (Sigma-Aldrich Co., St Louis, MO) at 41°C for 30 min according to Maroux et al. (1973). To determine activities of alkaline phosphatase, the 20 μl supernatant (2 times dilution) and serum (10 times diluted) were incubated with 180 μl 10 mM *p*-nitrophenyl phosphate solution at 41°C for 60 min according to Lackeyram et al. (2010). The absorbance of the end products (*p*-nitrophenyl and *p*-nitroanilide) was determined at 400 nm by using a spectrophotometer (VICTOR Nivo, Perkin Elmer, Pontyclun, United Kingdom) and quantify using a prepared standard curve. The activities of the enzymes except alkaline phosphatase in the serum were expressed as their values per mg protein per min. The activities of serum alkaline phosphatase were expressed as their values per mL serum per min.

### 2.6 RNA extraction and real-time reverse transcription-PCR analysis

Approximately 100 mg of mid-jejunum (whole tissue) samples were homogenized using a bead beater (Biospec Products, Bartlesville, OK) in QIAzol lysis reagents (Qiagen, Valencia, CA). Afterwards, RNA was extracted according to the manufacturer’s protocol. RNA quantity and quality were measured by a NanoDrop 2000 spectrophotometer (Thermo Fisher Scientific). One microgram of RNA was used to synthesize the first-strand cDNA by using high-capacity cDNA synthesis kits (Applied Biosystems, Foster City, CA) according to the manufacturer’s instructions. The 20 μl cDNA was diluted with 80 μl water. Primers used in the study are listed in Table 2. Real-time reverse transcript (RT)-PCR was conducted using SYBR Green Master Mix with a Step Onethermocycler (Applied Biosystem). The final volume for PCR mixture was 10 μl which included 5 μl of SYBR Green Master Mix (Applied Biosystems), 1.5 μl of cDNA, 0.5 μl of forward and reverse primers (10 μM each), and 2.5 μl of water. The thermal cycle condition for all genes was 95°C denature for 10 min, 40 cycles at 95°C for 15 s and 60°C for 1 min, 95°C for 15 s, 60°C for 1 min, and 95°C for 15 s. After the PCR amplification, melting curve analysis and product size verification by gel electrophoresis were conducted to check the specificity of the PCR reactions. The geometric mean of Ct values of glyceraldehyde 3-phosphate dehydrogenase (*GAPDH*) and beta-actin were used as reference values to normalize all target genes’ mRNA abundance (Vandesompele et al., 2002). Relative mRNA abundance of target genes was calculated using the 2^−ΔΔCT^ method, and the TA0 group was set as the control group (Livak and Schmittgen, 2001). Each sample was analyzed in duplicate, and the negative control, containing water instead of cDNA, was included in each run.

**TABLE 2:** Primers used in the study.

Genes	Sequence, 5′–3′	Amplicon	Accession number
*GAPDH*	F: GCT AAG GCT GTG GGG AAA GT	161	NM_204305.2
	R: TCA GCA GCA GCC TTC ACT AC		
*Beta actin*	F: CAA CAC AGT GCT GTC TGG TGG TA	205	NM_205518.2
	R: ATC GTA CTC CTG CTT GCT GAT CC		
*ZO2*	F: ATC CAA GAA GGC ACC TCA GC	100	NM_204918.1
	R: CAT CCT CCC GAA CAA TGC		
*CLDN2*	F: CCT GCT CAC CCT CAT TGG AG	145	NM_001277622.1
	R: GCT GAA CTC ACT CTT GGG CT		
*MUC2*	F: ATG CGA TGT TAA CAC AGG ACT C	110	JX284122.1
	R: GTG GAG CAC AGC AGA CTT TG		
*B0AT1*	F: GGG TTT TGT GTT GGC TTA GGA A	60	XM_419056.6
	R: TCC ATG GCT CTG GCA GAG AT		
*PepT1*	F: CCC CTG AGG AGG ATC ACT GTT	66	NM_204365.2
	R: CAA AAG AGC AGC AGC AAC GA		
*SGLT1*	F: GCC ATG GCC AGG GCT TA	71	NM_001293240.1
	R: CAA TAA CCT GAT CTG TGC ACC AGT A		
*EAAT3*	F: TGC TGC TTT GGA TTC CAG TGT	79	XM_424930.6
	R: AGC AAT GAC TGT AGT GCA GAA GTA ATA TAT G		

1 *GAPDH*, glyceraldehyde 3-phosphate dehydrogenase; *ZO2*, zonula occludens 2; *CLDN2*, claudin 2; *MUC2*, mucin 2; *B0AT1*, sodium-dependent neutral amino acid transporter 1; *PepT1*, peptide transporter 1; *SGLT1*, sodium glucose transporter 1; and *EAAT3*, excitatory amino acid transporter 3.

### 2.7 Liver total antioxidant capacity, concentrations of glutathione and oxidized glutathione, and activities of superoxide dismutase

Approximately 100 mg of liver samples were homogenized using a bead beater (Biospec Products, Bartlesville, OK) in selected solution for each assay. Afterwards, the samples were centrifuged at 12,000 × *g* for 15 min at 4°C, and protein concentration of the supernatants were analyzed using Pierce BCA protein assay kits (Thermo Fisher Scientific) after 20-time sample dilution. The total antioxidant capacity (TAC) of the collected supernatant was analyzed using a commercial kit (QuantiCromAntioxidant Assay Kit, BioAssay Systems, Hayward, CA) after 2-time sample dilution. Concentrations of glutathione (GSH) and oxidized glutathione (GSSG) in the supernatant were analyzed using Caymans GSH assay kits (Cayman Chemical, Ann Arbor, MI) with 20- and 2-time sample dilutions, respectively. The activities of superoxide dismutase (SOD) in the supernatants were determined Caymans SOD assay kits (Cayman Chemical) after 400-time sample dilution. The TAC, concentrations of GSH and GSSG, and SOD activities were expressed as values per mg protein.

### 2.8 DNA extraction and microbiome analysis

DNA was extracted from the cecal contents using QIAamp^®^ DNA stool mini kits (Qiagen GmbH, Hilden, Germany) according to manufacturer’s protocol. After quality and quantity of extracted DNA were checked using a NanoDrop 2000 spectrophotometer (Thermo Fisher Scientific), the samples were sent to LC sciences (Houston, TX) for 16 s rRNA gene sequencing (Choi et al., 2022d). Qimme2 (version 2022.02) was used to process and analyze 16s rRNA gene sequences (Bolyen et al., 2019). According to Choi and Kim (2022), 16s rRNA sequences were processed. The sampling depth for both D 18 and 36 time points was set as 45,000. By using Qiime2’s built-in functions, alpha diversity, beta diversity, and phylum and family level composition were analyzed and presented.

### 2.9 Slaughter, carcass processing, and breast myopathy evaluation

On D 42, three birds per pen were randomly selected from each pen for processing, and feed was removed from the pen for 12 h (Wang J. et al., 2020). On D 43, the selected birds were individually weighed and transferred to the processing plant at the University of Georgia. Birds were shackled, electrically stunned, bled, scalded, and defeathered. Following head and feet removal, the carcasses were eviscerated. Weights of the hot carcass and abdominal fat collected from fat around cloaca, bursa of Fabricius, gizzard, and proventriculus (Castro et al., 2019) were recorded. The carcasses were rinsed and chilled in ice-cold water at 1°C for 4 h. Legs, breast muscle, tender, wings, and skeleton were separated by trained personnel, and their weights were recorded. Breast myopathies and quality defects including white striping [score 0 (normal), 1, 2, and 3 (severe)], woody breast [score 1 (normal), 2, and 3 (severe)], spaghetti meat [score 0 (normal), 1, and 2 (severe)], and petechial hemorrhagic lesions [score 0 (normal), 1, 2, and 3 (severe)] were assessed by a trained expert according to published criteria (Kuttappan et al., 2017; Pang et al., 2020; Baldi et al., 2021; Prisco et al., 2021).

### 2.10 Breast meat quality measurements

Breast muscles from two birds per pen were stored at 1°C overnight for further meat quality analyses. Color and pH of the breast muscles were analyzed according to Brambila et al. (2018) with the modification. Color indicators including lightness (L*), redness (a*), and yellowness (b*) were determined in duplicate on the dorsal surface of breast meat by a Minolta Spectrophotometer CM-700 days (Konica Minolta Inc., Ramsey, NJ). Meat pH was analyzed (one measurement per fillet) by using a Thermo Scientific Orion Star™ A221 portable pH meter with a spear tipped probe (Thermal Scientific Orion 8163BNWP) (Thermo Fisher Scientific, Waltham, MA 02451, United States) that penetrated the cranial end of the intact breast muscle. Drip loss was analyzed by using a EZ-driploss method (Kaić et al., 2021). One cylindrical muscle core (2.5 cm diameter) was removed from the cranial side of the breast meat and trimmed to a similar height. The cores were weighed and placed in individual EZ containers (Danish Meat Research Institute, Taastrup, Denmark). The sealed containers were then stored in a refrigerator at 4°C. The samples were reweighted (approximately 7 g–8 g) after 48 h to determine drip loss (%). For thawing loss (%), intact breast samples were weighed and individually sealed in cooking bags before frozen at −20°C. The frozen samples were stored for 2 weeks at −20°C and were thawed at 4°C overnight and weighed again after liquid was removed. Cooking was performed by using a Henny Penny MCS-6 combi oven (Henny Penny Corp. Eaton, OH) on the Tender Steam setting at 84°C. Fillets were cooked ventral side up in stainless steel oven pans to an endpoint temperature of 74°C in the thickest part of the fillet (Brambila et al., 2018). A thermocouple system with hypodermic needle microprobes (Physitemp Instruments, Inc., Clifton, NJ) was used to monitor temperature. The samples were reweighed after the liquid was removed.

### 2.11 Statistical analyses

SAS (version 9.4; SAS Inst. Inc., Cary, NC) and GraphPad Prism (Version 9.1.0; GraphPad Software, San Diego, CA) were used for statistical analyses and graph construction. Treatment groups were compared using PROC MIXED followed by the Tukey’s individual comparison test. Orthogonal polynomial contrasts were conducted to assess the significance of linear or quadratic effects of the supplementation of TA in broilers. Pen was considered as the experimental unit, and values of individual birds in the same pen were averaged for meat analyses. Breast meat myopathy score and FPD score and incidence were analyzed using the Kruskal–Wallis test followed by the Dwass–Steel–Critchlow–Fligner *post hoc* test. Significance level was set at *p* < 0.05, and tendencies were also presented at 0.05 < *p* ≤ 0.10 (Choi et al., 2021).

## 3 Results

### 3.1 Growth performance

Results of the growth performance are presented in Table 3. In the starter phase, the TA2 group had significantly lower BW and ADG compared to the TA0 group, and the supplementation of TA linearly decreased BW and ADG in broiler chickens (*p* < 0.01). The ADFI was also linearly reduced by the supplementation of TA in broilers in the starter phase (*p* < 0.05). In the grower phase, the supplementation of TA linearly reduced BW and ADG and linearly and quadratically increased FCR of broilers (*p* < 0.01). The TA2 group had significantly lower BW compared to the TA0 group. The TA2 group had significantly lower ADG compared to the TA0 and TA1 groups (*p* < 0.05). The TA0.5 group had a significantly lower ADG compared to the TA0 (*p* < 0.05). The TA2 group had the highest FCR (*p* < 0.05) among the treatment groups, and the TA1 group had significantly lower FCR compared to the TA0.5 group. No statistical differences were observed in the growth performance parameters of the finisher phase (*p* > 0.1). In the whole phase, the supplementation of TA linearly increased FCR (*p* < 0.01), and the TA2 group had significantly higher FCR compared to the TA0 group.

**TABLE 3:** Growth performance parameters including body weight (BW, g), average daily gain (ADG, g/d), average daily feed intake (ADFI, g/d), and feed conversion ratio (FCR, g/g) in broilers fed diets supplemented with tannic acid on D 42^a^.

								Polynomial contrast^c^	
Items	TA0	TA0.25	TA0.5	TA1	TA2	SEM	*p*-value^b^	Linear	Quadratic
Initial BW, g	45.96	45.94	45.94	45.96	45.94	0.09	0.986		
Starter (D 0 to 18)									
BW	890.24^a^	860.97^a^ ^,^ ^b^	866.14^a^ ^,^ ^b^	853.03^a^ ^,^ ^b^	822.86^b^	38.63	0.045	0.003	0.787
ADG	46.90^a^	45.28^a^ ^,^ ^b^	45.57^a^ ^,^ ^b^	44.84^a^ ^,^ ^b^	43.16^b^	2.14	0.044	0.003	0.787
ADFI	59.79	58.09	58.39	57.27	56.64	2.47	0.187	0.030	0.411
FCR	1.28	1.28	1.28	1.28	1.31	0.04	0.469	0.108	0.479
Grower (D 18 to 28)									
BW	2020.4^a^	1959.23^a^ ^,^ ^b^	1943.7^a^ ^,^ ^b^	1973.35^a^ ^,^ ^b^	1892.55^b^	56.15	0.004	0.001	0.911
ADG	113.19^a^	109.65^a^ ^,^ ^b^ ^,^ ^c^	107.76^b^ ^,^ ^c^	112.03^a^ ^,^ ^b^	106.97^c^	2.89	0.001	0.007	0.948
ADFI	173.42	170.73	168.97	170.98	173.66	4.64	0.305	0.445	0.089
FCR	1.53^b^ ^,^ ^c^	1.56^b^ ^,^ ^c^	1.57^b^	1.53^c^	1.62^a^	0.03	< 0.001	<0.001	0.005
Finisher (D 28 to 42)									
BW	3,772.68	3,671.24	3,630.39	3,711.05	3,634.49	116.86	0.154	0.155	0.528
ADG	125.16	122.29	120.48	124.12	124.42	6.35	0.648	0.709	0.447
ADFI	214.79	212.52	214.21	216.75	218.59	10.86	0.857	0.323	0.927
FCR	1.72	1.74	1.78	1.75	1.76	0.07	0.616	0.507	0.413
Whole (D 0 to 42)									
ADG	88.77	86.28	85.34	87.26	85.44	2.77	0.143	0.152	0.514
ADFI	138.51	136.38	136.66	137.5	138.49	4.42	0.839	0.642	0.481
FCR	1.48^b^	1.50^a^ ^,^ ^b^	1.52^a^ ^,^ ^b^	1.49^a^ ^,^ ^b^	1.53^a^	0.03	0.023	0.008	0.713

^a^ TA0 (tannic acid 0; basal diet without TA); TA0.25 (tannic acid 0.25; basal diet + 0.25 g/kg TA); TA0.5 (tannic acid 0.5; basal diet + 0.5 g/kg TA); TA1 (tannic acid 1; basal diet + 1 g/kg TA); and TA2 (tannic acid 2; basal diet + 2 g/kg TA).

^b^ Treatment groups (7 replicates per treatment) were compared using PROC MIXED, followed by the Tukey’s individual comparison test. Different letters in the same row means significant differences (*p* < 0.05) among the treatments.

^c^ Orthogonal polynomial contrasts were conducted to assess the significance of linear or quadratic effects of the supplementation of TA, in broilers.

### 3.2 Intestinal morphology

As shown in Table 4, the TA0 group had significantly higher jejunal CD compared to the TA supplemented groups, and the supplementation of TA linearly (*p* < 0.01) and quadratically (*p* < 0.05) reduced jejunal CD on D 18. The TA0.5 and TA2 groups had greater jejunal VH:CD compared to the TA0 group (*p* < 0.05), and the supplementation of TA linearly increased jejunal VH:CD. The supplementation of TA tended to linearly decrease ileal VH (*p* = 0.075). The TA0.25 group tended to have lower ileal CD compared to the TA0 group (*p* = 0.057). The supplementation of TA quadratically decreased ileal CD and increased ileal VH:CD (*p* < 0.05). There were no statistical differences in the intestinal morphology on D 36 (*p* > 0.1).

**TABLE 4:** Duodenal, jejunal, and ileal morphology parameters including villus height (VH, µm), crypt depth (CD, µm), and VH:CD in broilers fed diets supplemented with tannic acid on D 18 and 36^a^.

								Polynomial contrast^3^	
Items	TA0	TA0.25	TA0.5	TA1	TA2	SEM	*p*-value^b^	Linear	Quadratic
D 18									
Duodenal VH	2,239.4	2,282.5	2,337.6	2,256.7	2,269.6	260.8	0.964	0.979	0.760
Duodenal CD	212	221.4	215.9	214.0	191	39.71	0.664	0.195	0.478
Duodenal VH:CD	11.23	11.18	11.08	10.69	12.21	1.86	0.637	0.305	0.268
Jejunal VH	1,240.7	1,151.5	1,218.1	1,205.7	1,198.8	194.83	0.937	0.926	0.890
Jejunal CD	286.36^a^	219.76^b^	203.72^b^	223.19^b^	200.78^b^	36.08	< 0.001	0.003	0.027
Jejunal VH:CD	4.55^b^	5.52^a^ ^,^ ^b^	6.29^a^	5.59^a^ ^,^ ^b^	6.26^a^	1.09	0.036	0.031	0.209
Ileal VH	916.94	805.3	910.39	856.22	791.58	103.75	0.096	0.075	0.745
Ileal CD	222.44^a^	173.9^b^	185.65^a^ ^,^ ^b^	184.37^a^ ^,^ ^b^	193.79^a^ ^,^ ^b^	30.29	0.057	0.517	0.046
Ileal VH:CD	4.26	4.88	5.10	4.85	4.26	0.815	0.212	0.451	0.046
D 36									
Duodenal VH	2,596.9	2,718.2	2,582.5	2,608.2	2,849.4	424.4	0.737	0.311	0.522
Duodenal CD	206.66	201.69	172.23	198.67	204.99	33.85	0.325	0.731	0.224
Duodenal VH:CD	13.09	14.62	15.91	13.70	14.56	2.93	0.459	0.768	0.536
Jejunal VH	1,543.5	1781.9	1,410.2	1795.8	1758	248.96	0.042	0.109	0.796
Jejunal CD	179.9	186.74	163.79	175.59	193.21	28.03	0.366	0.342	0.201
Jejunal VH:CD	9.11	10	8.89	11.24	9.4	1.88	0.165	0.661	0.113
Ileal VH	1,050.5	912.5	958.5	1,010.1	1,016.7	147.93	0.454	0.657	0.496
Ileal CD	157.47	155.29	149.88	149.77	157.91	24.36	0.943	0.912	0.412
Ileal VH:CD	6.96	6.32	6.78	7.04	6.68	1.28	0.849	0.971	0.795

^a^ TA0 (tannic acid 0; basal diet without TA); TA0.25 (tannic acid 0.25; basal diet + 0.25 g/kg TA); TA0.5 (tannic acid 0.5; basal diet + 0.5 g/kg TA); TA1 (tannic acid 1; basal diet + 1 g/kg TA); and TA2 (tannic acid 2; basal diet + 2 g/kg TA).

^b^ Treatment groups (7 replicates per treatment) were compared using PROC MIXED, followed by the Tukey’s individual comparison test. Different letters in the same row means significant differences (*p* < 0.05) among the treatments.

^c^ Orthogonal polynomial contrasts were conducted to assess the significance of linear or quadratic effects of the supplementation of TA in broilers.

### 3.3 Activities of jejunal brush border digestive enzymes and serum alkaline phosphatase

As shown in Table 5, the supplementation of TA quadratically increased sucrase activities (*p* < 0.05) and quadratically decreased lipase activities in the jejunum tissue (*p* < 0.05). On D 36, jejunal lipase activities were quadratically decreased by the supplementation of TA (*p* < 0.05). However, no differences were observed in the activities of jejunal sucrase, LAP, intestinal alkaline phosphatase (IAP), and serum alkaline phosphatase (SAP) (*p* > 0.1).

**TABLE 5:** Activities of jejunal brush border digestive enzymes including maltase (nmol glucose released/mg protein/min), sucrase (nmol glucose released/mg protein/min), leucine aminopeptidase (LAP; nmol p-nitroaniline liberated/mg protein/min), intestinal alkaline phosphatase (IAP; μmol p-nitrophenol liberated/mg protein/min), lipase (mmol p-nitrophenyl phosphate liberated/mg protein/min), and serum alkaline phosphatase (SAP; μmol p-nitrophenol liberated/ml serum/min) in broilers fed diets supplemented with tannic acid on D 18 and 36^a^.

								Polynomial contrast^c^	
Items	TA0	TA0.25	TA0.5	TA1	TA2	SEM	*p*-value^b^	Linear	Quadratic
D 18									
Maltase	0.218	0.247	0.227	0.233	0.232	0.066	0.951	0.906	0.826
Sucrase	0.162	0.277	0.334	0.315	0.243	0.136	0.170	0.634	0.027
LAP	25.41	25.88	20.55	25.84	22.27	6.23	0.388	0.452	0.968
Lipase	1.612	1.138	0.751	0.861	1.212	0.623	0.113	0.533	0.016
IAP	0.206	0.241	0.196	0.213	0.219	0.053	0.596	0.912	0.775
SAP	0.23	0.26	0.25	0.23	0.26	0.06	0.844	0.634	0.791
D 36									
Maltase	2.221	1.809	1.876	1.750	1.815	0.681	0.713	0.421	0.353
Sucrase	0.453	0.497	0.426	0.326	0.408	0.209	0.639	0.417	0.350
LAP	12.77	13.67	11.37	11.64	12.71	2.65	0.495	0.775	0.236
Lipase	1.250	1.148	1.115	0.996	1.244	0.249	0.316	0.930	0.038
IAP	0.254	0.238	0.235	0.256	0.271	0.044	0.544	0.199	0.492
SAP	0.21	0.20	0.20	0.18	0.2	0.04	0.680	0.442	0.226

^a^ TA0 (tannic acid 0; basal diet without TA); TA0.25 (tannic acid 0.25; basal diet + 0.25 g/kg TA); TA0.5 (tannic acid 0.5; basal diet + 0.5 g/kg TA); TA1 (tannic acid 1; basal diet + 1 g/kg TA); and TA2 (tannic acid 2; basal diet + 2 g/kg TA).

^b^ Treatment groups (7 replicates per treatment) were compared using PROC MIXED, followed by the Tukey’s individual comparison test.

^c^ Orthogonal polynomial contrasts were conducted to assess the significance of linear or quadratic effects of the supplementation of TA in broilers.

### 3.4 Apparent ileal digestibility of dry matter, organic matter, ash, crude protein, and crude fat

As shown in Table 6, the supplementation of TA linearly (*p* < 0.01) and quadratically (*p* < 0.05) increased AID of DM, OM, and CP on D 36. The TA0.25, TA0.5, TA1, and TA2 groups had significantly higher AID of DM compared to the TA0 group (*p* < 0.01). The AID of OM was significantly lower in the TA0 group compared to the TA0.25, TA0.5, TA1, and TA2 groups (*p* < 0.05). The TA2 group had significantly AID of ash compared to the TA0 group, and the supplementation of TA linearly increased AID of ash (*p* < 0.01). The TA0 group had the lowest AID of CP among the treatments (*p* < 0.05). No differences were observed in the AID of CF among the treatments (*p* > 0.1).

**TABLE 6:** Apparent ileal digestibility (%) of dry matter (DM), organic matter (DM), organic matter (OM), ash, crude protein (CP), and crude fat (CF) in broilers fed diets supplemented with tannic acid on D 18 and 36^a^.

								Polynomial contrast^c^	
Items	TA0	TA0.25	TA0.5	TA1	TA2	SEM	*p*-value^b^	Linear	Quadratic
DM	74.37^b^	81.00^a^	79.26^a^	79.24^a^	80.68^a^	2.39	<0.01	0.003	0.033
OM	76.10^b^	82.65^a^	80.83^a^	80.71^a^	82.12^a^	2.22	<0.01	0.003	0.03
Ash	40.39^b^	49.57^a^ ^,^ ^b^	48.86^a^ ^,^ ^b^	50.60^a^ ^,^ ^b^	52.79^a^	6.75	0.019	0.008	0.115
CP	80.57^b^	87.35^a^	85.08^a^	85.19^a^	86.62^a^	2.09	<0.01	0.002	0.028
CF	84.62	90.41	85.82	85.98	87.01	4.52	0.185	0.962	0.948

^a^ TA0 (tannic acid 0; basal diet without TA); TA0.25 (tannic acid 0.25; basal diet + 0.25 g/kg TA); TA0.5 (tannic acid 0.5; basal diet + 0.5 g/kg TA); TA1 (tannic acid 1; basal diet + 1 g/kg TA); and TA2 (tannic acid 2; basal diet + 2 g/kg TA).

^b^ Treatment groups (7 replicates per treatment) were compared using PROC MIXED, followed by the Tukey’s individual comparison test. Different letters in the same row means significant differences (*p* < 0.05) among the treatments.

^c^ Orthogonal polynomial contrasts were conducted to assess the significance of linear or quadratic effects of the supplementation of TA in broilers.

### 3.5 Relative mRNA expression of genes related tight junction proteins and nutrient transporters in the jejunum

Relative mRNA expression of genes related to tight junction proteins and nutrient transporters in the jejunum is presented in Table 7. On D 18, the TA1 group had significantly higher relative mRNA expression of zonula occludens 2 (*Z O 2*) compared to the TA0, TA0.25 and TA0.5 groups, and the supplementation of TA quadratically increased relative mRNA expression of *Z O 2* (*p* < 0.05). The supplementation of TA linearly increased relative mRNA expression of claudin 2 (*CLDN2*; *p* < 0.01) and junctional adhesion molecule 2 (*JAM2*; *p* < 0.05). The TA2 group had significantly higher relative mRNA expression of mucin 2 (*MUC2*) compared to the TA0.25 group (*p* < 0.05), and the supplementation of TA linearly increase relative mRNA expression of *MUC2* (*p* < 0.05). The TA1 group had significantly higher relative mRNA expression of sodium-dependent neutral amino acid transporter (*B0AT1*) compared to the TA0.25 group (*p* < 0.05), and the supplementation of TA linearly increase relative mRNA expression of *B0AT1* (*p* < 0.05). The supplementation of TA tended to modulate (*p* = 0.061) and quadratically increased relative mRNA expression of peptide transporter 1 (*PepT1*) (*p* < 0.05). The TA1 and TA2 groups had significantly higher relative mRNA expression of sodium glucose cotransporter 1 (*SGLT1*) compared to the TA0.25 group, and the supplementation of TA linearly increased relative mRNA expression of *SGLT1* (*p* < 0.01). The TA1 group had significantly higher relative mRNA expression of excitatory amino acid transporter 3 (*EAAT3*) compared to the TA0, TA0.25, and TA0.5 groups, and the supplementation of TA quadratically increased relative mRNA expression of *EAAT3* (*p* < 0.05). The supplementation of TA tended to modulate (*p* = 0.064) and quadratically increased relative mRNA expression of mucin 2 (*MUC2*) on D 36 (*p* < 0.05). However, no differences in relative mRNA expression of tight junction proteins and nutrient transporters were observed among the treatments on D 36 (*p* > 0.1).

**TABLE 7:** Relative mRNA expression of gene associated with tight junction proteins and nutrients transporters in broilers fed diets supplemented with tannic acid on D 18 and 36^a^.

								Polynomial contrast^c^	
Items	TA0	TA0.25	TA0.5	TA1	TA2	SEM	*p*-value^b^	Linear	Quadratic
D 18									
*Z O 2*	1.11^b^	1.11^b^	1.14^b^	1.93^a^	1.31^a^ ^,^ ^b^	0.51	0.021	0.148	0.022
*CLDN2*	1.08	0.87	1.34	1.59	2.09	0.86	0.103	0.009	0.94
*JAM2*	1.42	0.84	0.72	1.03	4.6	3.23	0.16	0.035	0.171
*MUC2*	1.16^a^ ^,^ ^b^	0.76^b^	1.01^a^ ^,^ ^b^	1.47^a^ ^,^ ^b^	1.79^a^	0.56	0.017	0.003	0.67
*B0AT1*	1.06^a^ ^,^ ^b^	0.54^b^	0.91^a^ ^,^ ^b^	1.84^a^	1.38^a^ ^,^ ^b^	0.69	0.017	0.039	0.195
*PepT1*	1.12	0.9	1.29	2.03	1.32	0.71	0.061	0.203	0.038
*SGLT1*	1.14^a^ ^,^ ^b^	0.77^b^	0.93^a^ ^,^ ^b^	1.65^a^	1.76^a^	0.54	0.005	0.001	0.894
*EAAT3*	1.11^b^	1.11^b^	1.14^b^	1.93^a^	1.31^a^ ^,^ ^b^	0.51	0.022	0.148	0.022
D 36									
*Z O 2*	1.14	2.03	1.49	1.7	1.48	1.04	0.606	0.95	0.451
*CLDN2*	1.84	1.55	1.26	0.98	1.51	1.84	0.926	0.762	0.385
*JAM2*	1.59	1.44	5.44	2.76	2.67	3.18	0.158	0.692	0.187
*MUC2*	1.02	1.12	1.56	1.83	1.43	0.53	0.064	0.116	0.015
*B0AT1*	1.09	0.97	1.07	1.54	1.18	0.71	0.233	0.313	0.17
*PepT1*	1.59	0.8	1.78	1.38	1.45	1.4	0.748	0.885	0.979
*SGLT1*	1.1	1.21	1.49	1.69	1.55	0.59	0.331	0.130	0.146
*EAAT3*	2.3	1.8	1.93	1.02	1.19	2	0.741	0.262	0.524

^a^ TA0 (tannic acid 0; basal diet without TA); TA0.25 (tannic acid 0.25; basal diet + 0.25 g/kg TA); TA0.5 (tannic acid 0.5; basal diet + 0.5 g/kg TA); TA1 (tannic acid 1; basal diet + 1 g/kg TA); and TA2 (tannic acid 2; basal diet + 2 g/kg TA). *Z O 2*, zonula occludens 2; *CLDN2*, claudin 2; *MUC2*, mucin 2; *B0AT1*, sodium-dependent neutral amino acid transporter 1; *PepT1*, peptide transporter 1; *SGLT1*, sodium glucose transporter 1; and *EAAT3*, excitatory amino acid transporter 3.

^b^ Treatment groups (7 replicates per treatment) were compared using PROC MIXED, followed by the Tukey’s individual comparison test. Different letters in the same row means significant differences (*p* < 0.05) among the treatments.

^c^ Orthogonal polynomial contrasts were conducted to assess the significance of linear or quadratic effects of the supplementation of TA in broilers.

### 3.6 Liver total antioxidant capacity, concentrations of glutathione and oxidized glutathione, and activities of superoxide dismutase

The supplementation of TA tended to modulate TAC in the liver on D 36 (*p* = 0.073). No differences were observed in concentrations of GSH and GSSG and activities of SOD in the liver on D 18 and 36 (Table 8; *p* > 0.1).

**TABLE 8:** Total antioxidant capacity (TAC; µM Trolox Equivalents/mg protein), concentrations of glutathione (GSH; µM/mg protein) and oxidized glutathione (GSSG; µM/mg protein), and activities of superoxide dismutase (SOD; U/mg protein) in broilers fed diets supplemented with tannic acid on D 18 and 36^a^.

								Polynomial contrast^c^	
Items	TA0	TA0.25	TA0.5	TA1	TA2	SEM	*p*-value^b^	Linear	Quadratic
D 18									
TAC	78.90	80.51	76.48	80.28	78.23	4.29	0.923	0.898	0.929
GSH	15.36	17.17	14.14	13.69	15.16	3.06	0.271	0.491	0.210
GSSG	2.07	2.25	1.8	1.86	1.96	0.51	0.509	0.494	0.337
Reduced GSH	11.22	12.67	10.54	9.97	11.24	2.17	0.219	0.517	0.188
Reduced GSH: GSSG	5.64	5.67	6.05	5.37	5.93	0.92	0.676	0.753	0.650
SOD	23.04	22.27	20.91	23.7	22.7	6.93	0.958	0.874	0.964
D 36									
TAC	90.31	80.65	77.94	81.93	79.12	8.38	0.073	0.114	0.146
GSH	17.11	15.83	14.37	16.20	15.96	4.3	0.828	0.896	0.543
GSSG	1.87	1.84	1.59	1.79	1.80	0.59	0.931	0.954	0.636
Reduced GSH	12.91	12.14	11.21	12.61	11.58	3.33	0.891	0.667	0.881
Reduced GSH: GSSG	7.01	7.02	7.16	7.25	6.54	1.33	0.902	0.512	0.461
SOD	24.34	25.57	25.51	25.24	25.5	8.29	0.998	0.879	0.883

^a^ TA0 (tannic acid 0; basal diet without TA); TA0.25 (tannic acid 0.25; basal diet + 0.25 g/kg TA); TA0.5 (tannic acid 0.5; basal diet + 0.5 g/kg TA); TA1 (tannic acid 1; basal diet + 1 g/kg TA); and TA2 (tannic acid 2; basal diet + 2 g/kg TA).

^b^ Treatment groups (7 replicates per treatment) were compared using PROC MIXED, followed by the Tukey’s individual comparison test. Different letters in the same row means significant differences (*p* < 0.05) among the treatments.

^c^ Orthogonal polynomial contrasts were conducted to assess the significance of linear or quadratic effects of the supplementation of TA in broilers.

### 3.7 Alpha diversity in the cecal bacterial communities

**FIGURE 1:**
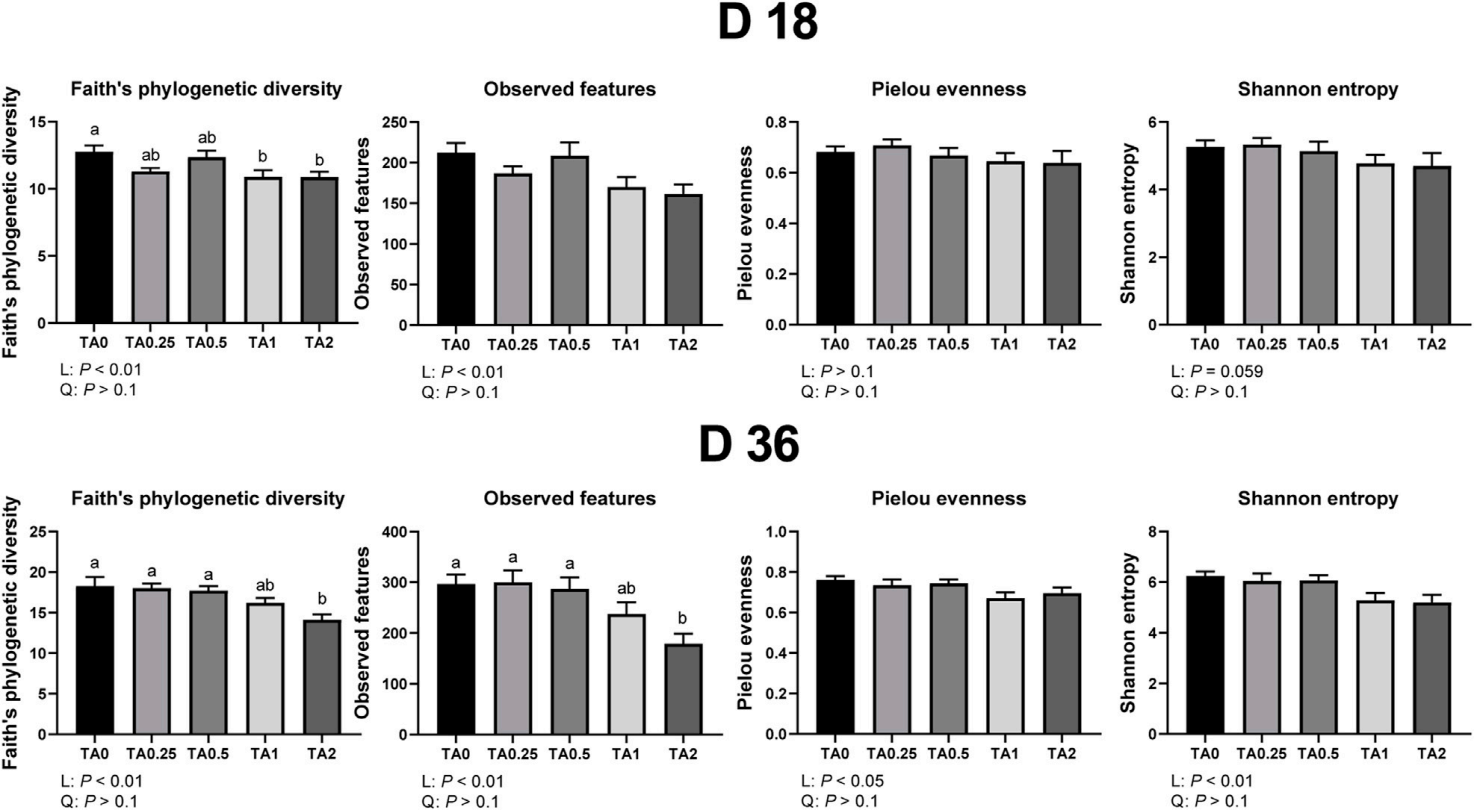
Alpha diversity parameters of the cecal microbial communities in the TA0 (tannic acid 0; basal diet without TA), TA0.25 (tannic acid 0.25; basal diet + 0.25 g/kg TA), TA0.5 (tannic acid 0.5; basal diet + 0.5 g/kg TA), TA1 (tannic acid 1; basal diet + 1 g/kg TA), and TA2 (tannic acid 2; basal diet + 2 g/kg TA) groups on D 18 and 36. All treatment groups (7 replicates per treatment) were compared using PROC MIXED followed by the Tukey’s individual comparison test. Different letters in the same row means significant differences (*p* < 0.05) among the treatments. Orthogonal polynomial contrasts were used to evaluate the significance of linear or quadratic effects of the supplementation of TA in broilers.

Alpha diversity indices including faith’s phylogenetic diversity, observed features, pielou evenness, and shannon entropy in the cecal bacterial communities on D 18 and 36 are shown in Figure 1. On D 18, the TA1 and TA2 groups had significantly lower faith’s phylogenetic diversity (communities’ evolutionary distance) compared to the TA0 group (*p* < 0.05), and the supplementation of TA linearly reduced faith’s phylogenetic diversity and observed features (richnesss) in the cecal bacterial communities (*p* < 0.05). The supplementation of TA tended to linearly reduce shannon entropy (richness and evenness) in the cecal bacterial communities (*p* = 0.059). On D 36, the TA2 group had lower faith’s phylogenetic diversity and observed features compared to TA0, TA0.25, and TA0.5 groups. The supplementation of TA linearly reduced faith’s phylogenetic diversity (*p* < 0.01), observed features (*p* < 0.01), pielou evenness (evenness; *p* < 0.05), and shannon entropy (*p* < 0.01) in the cecal bacterial communities.

### 3.8 Beta diversity in the cecal bacterial communities

**FIGURE 2:**
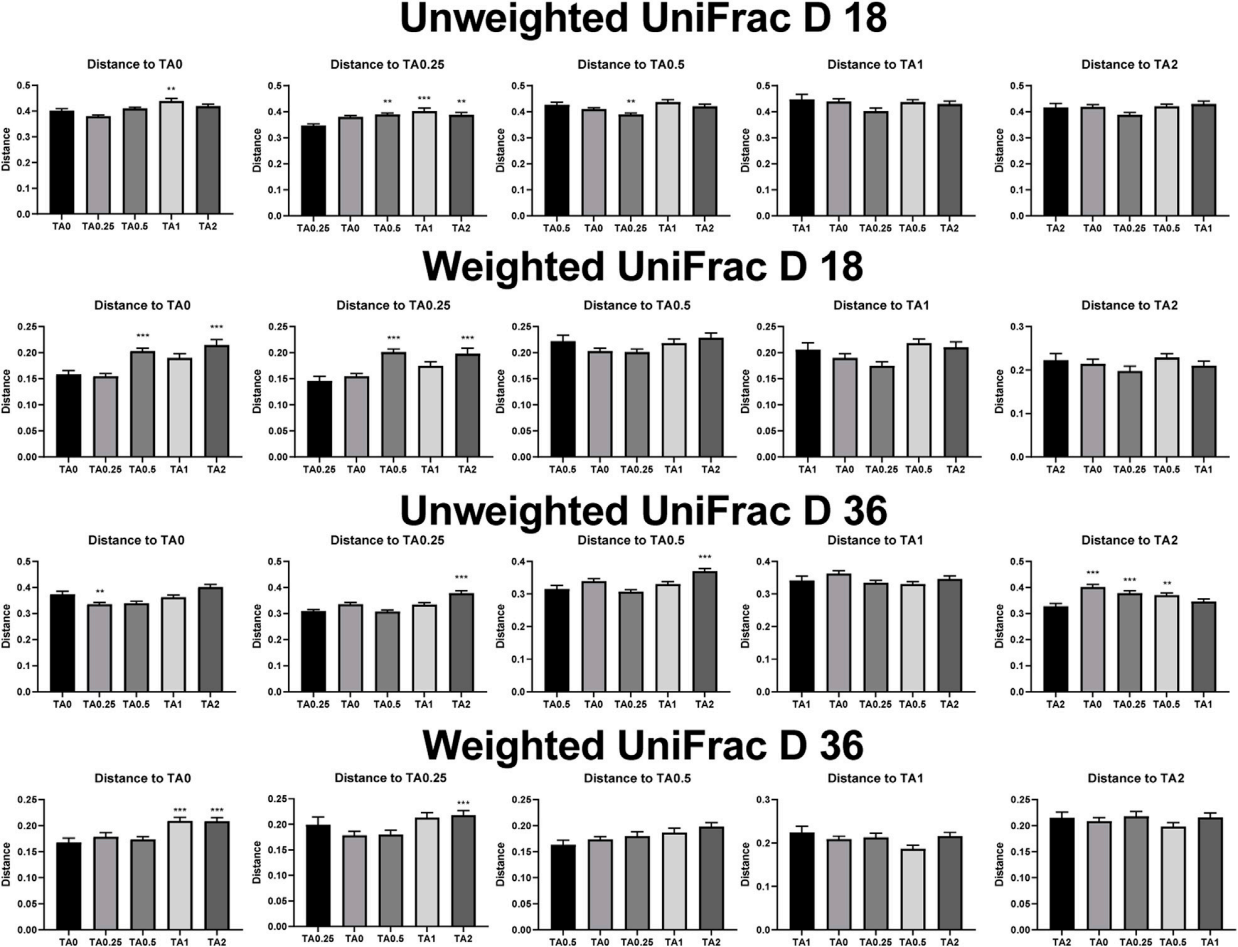
Beta diversity indices including unweighted and weighted unifrac of the cecal microbial communities in the TA0 (tannic acid 0; basal diet without TA), TA0.25 (tannic acid 0.25; basal diet + 0.25 g/kg TA), TA0.5 (tannic acid 0.5; basal diet + 0.5 g/kg TA), TA1 (tannic acid 1; basal diet + 1 g/kg TA), and TA2 (tannic acid 2; basal diet + 2 g/kg TA) groups on D 18 and 36. All treatment groups (7 replicates per treatment) were compared using PROC MIXED followed by the Tukey’s individual comparison test. Orthogonal polynomial contrasts were used to evaluate the significance of linear or quadratic effects of the supplementation of TA in broilers.

**FIGURE 3:**
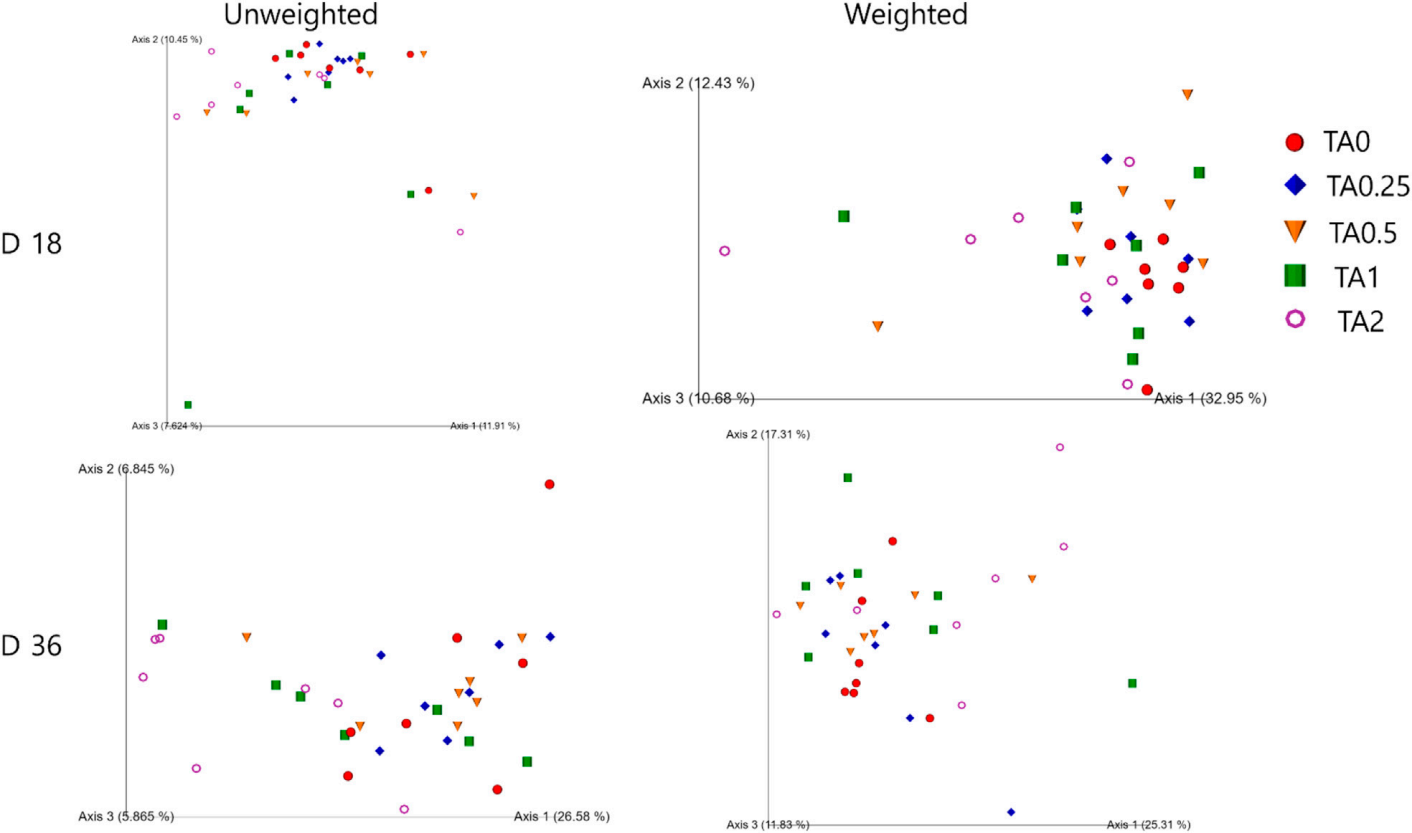
Visualized beta diversity parameters including unweighted and weighted unifrac of the cecal microbial communities in the TA0 (tannic acid 0; basal diet without TA), TA0.25 (tannic acid 0.25; basal diet + 0.25 g/kg TA), TA0.5 (tannic acid 0.5; basal diet + 0.5 g/kg TA), TA1 (tannic acid 1; basal diet + 1 g/kg TA), and TA2 (tannic acid 2; basal diet + 2 g/kg TA) groups on D 18 and 36. All treatment groups (7 replicates per treatment) were compared using PROC MIXED followed by the Tukey’s individual comparison test. Orthogonal polynomial contrasts were used to evaluate the significance of linear or quadratic effects of the supplementation of TA in broilers.

As shown in Figure 2, the TA1 group had significantly greater unweighted unifrac distance (the sum of the branch length without considering bacterial abundance) compared to the TA0 group on D 18. The TA0.5, TA1, and TA2 groups had significantly greater unweighted unifrac distance compared to the TA0.25 group. The TA0.25 group had significantly greater unweighted unifrac distance compared to the TA0.5 group (*p* < 0.05). The TA0.5 and TA2 groups had significantly greater weighted unifrac distance (the sum of the branch length with considering bacterial abundance) compared to the TA0 group. The TA0.5 and TA2 groups had significantly greater weighted unifrac distance compared to the TA0.25 group. On D 36, TA0.25 had significantly lower unweighted unifrac distance compared to the TA0 group. The TA2 group had significantly greater unweighted unifrac distance compared to the TA0.25 and TA0.5 groups. The TA0, TA0.25, and TA0.5 groups had significantly higher unweighted unifrac distance compared to the TA2 group. The TA1 and TA2 groups had significantly greater weighted unifrac distance compared to the TA0 group. The TA2 group had significantly higher weighted unifrac distance compared to the TA0.25 group. However, no visual differences were observed in the beta diversity indices including weighted and unweighted emperor on D 18 and 36 (Figure 3).

### 3.9 Bacterial composition in the cecal bacterial communities

**FIGURE 4:**
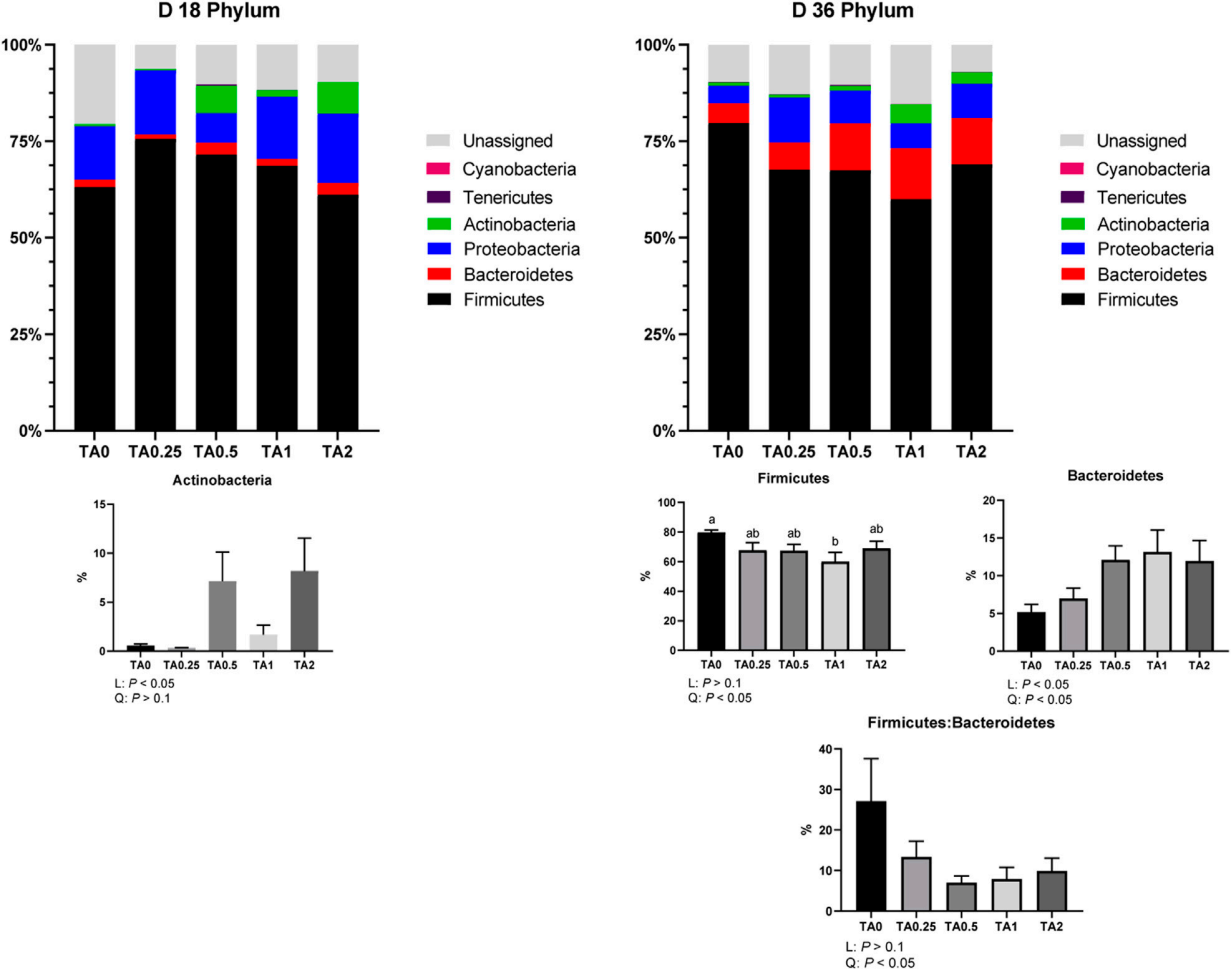
Phylum-level composition of the cecal microbial communities in the TA0 (tannic acid 0; basal diet without TA), TA0.25 (tannic acid 0.25; basal diet + 0.25 g/kg TA), TA0.5 (tannic acid 0.5; basal diet + 0.5 g/kg TA), TA1 (tannic acid 1; basal diet + 1 g/kg TA), and TA2 (tannic acid 2; basal diet + 2 g/kg TA) groups on D 18 and 36. All treatment groups (7 replicates per treatment) were compared using PROC MIXED followed by the Tukey’s individual comparison test. Orthogonal polynomial contrasts were used to evaluate the significance of linear or quadratic effects of the supplementation of TA in broilers.

As shown in Figure 4, the relative abundance of the phylum Actinobacteria was linearly increased by the supplementation of TA on D 18. On D 36, the relative abundance of the phylum Firmicutes was quadratically reduced by the supplementation of TA, and the TA1 group had significantly lower relative abundance of the phylum Firmicutes compared to the TA0 group. The relative abundance of the phylum Bacteroidetes was linearly (*p* < 0.05) and quadratically (*p* < 0.05) increased by the supplementation of TA. The supplementation of TA quadratically decreased the ratio of the phyla Firmicutes and Bacteroidetes (*p* < 0.05).

**FIGURE 5:**
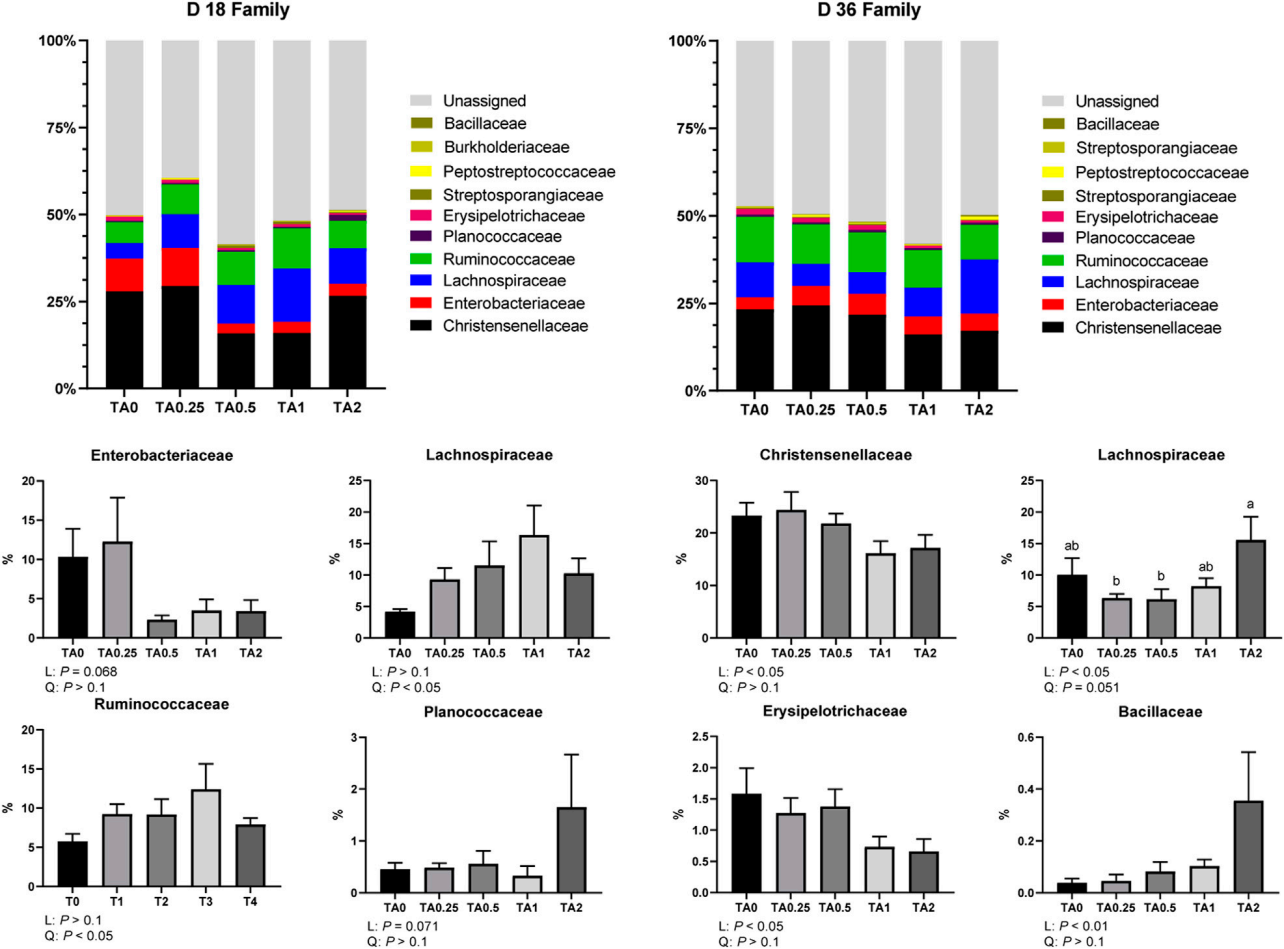
Family-level composition of the cecal microbial communities in the TA0 (tannic acid 0; basal diet without TA), TA0.25 (tannic acid 0.25; basal diet + 0.25 g/kg TA), TA0.5 (tannic acid 0.5; basal diet + 0.5 g/kg TA), TA1 (tannic acid 1; basal diet + 1 g/kg TA), and TA2 (tannic acid 2; basal diet + 2 g/kg TA) groups on D 18 and 36. All treatment groups (7 replicates per treatment) were compared using PROC MIXED followed by the Tukey’s individual comparison test. Orthogonal polynomial contrasts were used to evaluate the significance of linear or quadratic effects of the supplementation of TA in broilers.

As shown in Figure 5, the supplementation of TA tended to linearly reduce the relative abundance of the family Enterobacteriaceae (*p* = 0.068) and tended to linearly increase the relative abundance of the family Planococcaceae (*p* = 0.071) on D 18. The relative abundance of the families Lachnospiraceae and Ruminococcaceae was quadratically increased by the supplementation of TA. On D 36, the supplementation of TA linearly decreased the relative abundance of the families Christensenellaceae and Erysipelotrichaceae (*p* < 0.05). The supplementation of TA linearly increased the relative abundance of the family Bacillaceae (*p* < 0.01). The supplementation of TA linearly increased the relative abundance of the family Lachnospiraceae (*p* < 0.05) and tended to quadratically increased the relative abundance of the family Lachnospiraceae (*p* = 0.051). The TA2 group had significantly higher relative abundance of the family Lachnospiraceae compared to the TA0.25 and TA0.5 groups.

### 3.10 Litter ammonia concentration and foot pad dermatitis

**FIGURE 6:**
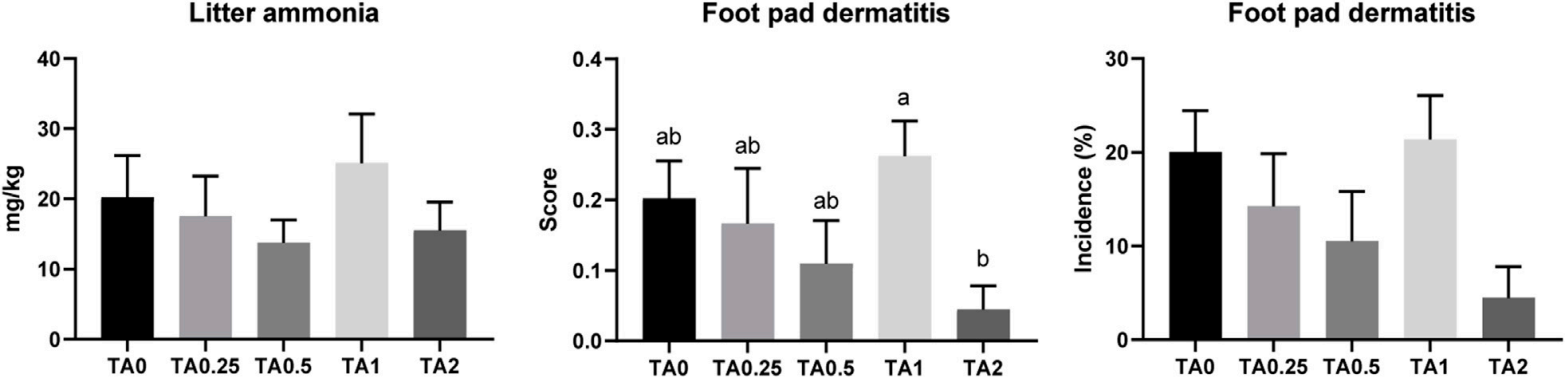
Litter ammonia concentration (mg/kg) and foot pad dermatitis score and incidence (%) in the TA0 (tannic acid 0; basal diet without TA), TA0.25 (tannic acid 0.25; basal diet + 0.25 g/kg TA), TA0.5 (tannic acid 0.5; basal diet + 0.5 g/kg TA), TA1 (tannic acid 1; basal diet + 1 g/kg TA), and TA2 (tannic acid 2; basal diet + 2 g/kg TA) groups on D 42. For litter ammonia concentration, all treatment groups (7 replicates per treatment) were compared using PROC MIXED followed by the Tukey’s individual comparison test, and different letters in the same row means significant differences (*p* < 0.05) among the treatments. Orthogonal polynomial contrasts were used to evaluate the significance of linear or quadratic effects of the supplementation of TA. Foot pad dermatitis score were analyzed using the Kruskal–Wallis test followed by the Dwass–Steel–Critchlow–Fligner *post hoc* test.

There were no differences in litter ammonia concentrations among the treatments on D 42 (Figure 6). The TA1 group had a significantly higher FPD score compared to the TA2 group on D 42. However, no differences were observed in the incidence of FPD among the treatments on D 42 (*p* > 0.1).

### 3.11 Bone health parameters and body composition

The supplementation of TA linearly reduced BMD (*p* < 0.01) and BMC (*p* < 0.05), and the TA2 group tended to have lower BMD (*p* = 0.051) and had significantly lower BMC (*p* < 0.05) compared to the TA0 group (*p* < 0.05) on D 42 (Table 9). The body fat percentage was linearly increased by the supplementation of TA (*p* < 0.05), and the supplementation of TA tended to reduce lean weight (*p* = 0.065). The lean:fat was linearly reduced by the supplementation of TA (*p* < 0.05).

**TABLE 9:** Bone health parameters including bone mineral content (BMC; g), bone mineral density (BMD; g/cm^2^) and body composition parameters including tissue weight (g), lean weight (g), fat weight (g), body fat percentage (%), and lean:fat (g/g) in broilers fed diets supplemented with tannic acid on D 42.

								Polynomial contrast^c^	
Items	TA0	TA0.25	TA0.5	TA1	TA2	SEM	*p*-value^b^	Linear	Quadratic
BMC	585.71^a^	546.57^a^ ^,^ ^b^	508.29^a^ ^,^ ^b^	525.43^a^ ^,^ ^b^	464.86^b^	59.76	0.011	0.001	0.516
BMD	216.14^a^	204^a^ ^,^ ^b^	206.43^a^ ^,^ ^b^	205.71^a^ ^,^ ^b^	199.71^b^	9.97	0.051	0.017	0.428
Tissue weight	3,668.44	3,444.03	3,388.12	3,462.08	3,304.22	359.7	0.430	0.141	0.596
Fat	668.38	644.74	677.21	712.4	686.23	84.33	0.663	0.401	0.435
Fat percentage	18.39	18.81	19.93	20.79	20.73	2.02	0.113	0.022	0.155
Lean weight	2,980.71	2,799.29	2,710.91	2,749.55	2,616.1	310.87	0.285	0.065	0.465
Lean:Fat	4.5	4.38	4.02	3.9	3.84	0.55	0.115	0.021	0.194

^a^ TA0 (tannic acid 0; basal diet without TA); TA0.25 (tannic acid 0.25; basal diet + 0.25 g/kg TA); TA0.5 (tannic acid 0.5; basal diet + 0.5 g/kg TA); TA1 (tannic acid 1; basal diet + 1 g/kg TA); and TA2 (tannic acid 2; basal diet + 2 g/kg TA).

^b^ Treatment groups (7 replicates per treatment) were compared using PROC MIXED, followed by the Tukey’s individual comparison test. Different letters in the same row means significant differences (*p* < 0.05) among the treatments.

^c^ Orthogonal polynomial contrasts were conducted to assess the significance of linear or quadratic effects of the supplementation of TA in broilers.

### 3.12 Hot weight, abdominal fat, chilled weight, and meat yield

On D 43, hot weight was linearly decreased by the supplementation of TA (*p* < 0.05) (Table 10). The supplementation of TA tended to linearly increase abdominal fat weight (*p* = 0.077) and linearly increased abdominal fat percentage (*p* < 0.05). Proportion of leg weight was increased by the supplementation of TA (*p* < 0.05).

**TABLE 10:** Hot weight, abdominal fat (g and %) weight and meat yield in broilers fed diets supplemented with tannic acid on D 43^a^.

								Polynomial contrast^c^	
Items	TA0	TA0.25	TA0.5	TA1	TA2	SEM	*p*-value^b^	Linear	Quadratic
Hot weight (g)	2,890.9	2,873.5	2,798.0	2,841.7	2,792.6	124	0.488	0.174	0.632
Abdominal fat (g)	42.10	40.48	42.67	40.05	48.29	7.21	0.237	0.077	0.197
Abdominal fat (%)	1.45	1.41	1.52	1.41	1.73	0.23	0.072	0.018	0.189
Total chilled weight	2,931.1	2,899.3	2,828.1	2,865.0	2,817.5	129.5	0.449	0.142	0.539
Legs (%)	26.92^b^	27.36^a^ ^,^ ^b^	27.95^a^ ^,^ ^b^	27.52^a^ ^,^ ^b^	28.40^a^	0.92	0.053	0.010	0.736
Breast (%)	27.22	26.29	26.33	26.15	26.00	1.30	0.457	0.168	0.350
Tender (%)	5.10	4.91	4.78	4.95	4.91	0.37	0.633	0.635	0.407
Wings (%)	9.73	9.80	10.05	9.89	9.96	0.37	0.525	0.349	0.400
Skeleton (%)	31.04	31.64	30.89	31.49	30.74	1.48	0.751	0.557	0.541

^a^ TA0 (tannic acid 0; basal diet without TA); TA0.25 (tannic acid 0.25; basal diet + 0.25 g/kg TA); TA0.5 (tannic acid 0.5; basal diet + 0.5 g/kg TA); TA1 (tannic acid 1; basal diet + 1 g/kg TA); and TA2 (tannic acid 2; basal diet + 2 g/kg TA).

^b^ Treatment groups (7 replicates per treatment) were compared using PROC MIXED, followed by the Tukey’s individual comparison test.

^c^ Orthogonal polynomial contrasts were conducted to assess the significance of linear or quadratic effects of the supplementation of TA in broilers.

### 3.13 Breast muscle myopathies and meat color (L*, a*, b*), pH, drip loss, thawing loss, and cooking loss in the breast meat

**FIGURE 7:**
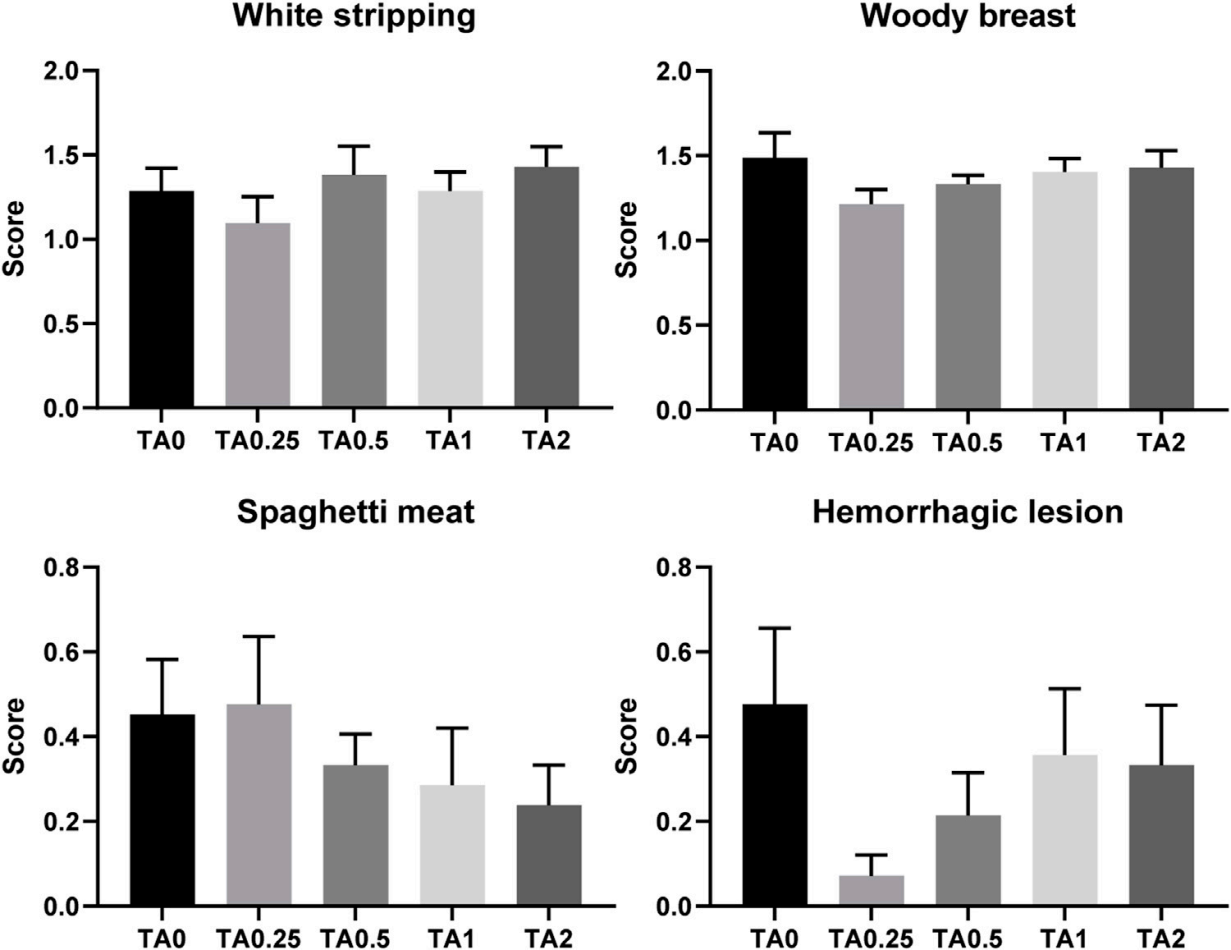
Breast muscle myopathies including white striping, woody breast, spaghetti meat, and hemorrhagic lesion in the TA0 (tannic acid 0; basal diet without TA), TA0.25 (tannic acid 0.25; basal diet + 0.25 g/kg TA), TA0.5 (tannic acid 0.5; basal diet + 0.5 g/kg TA), TA1 (tannic acid 1; basal diet + 1 g/kg TA), and TA2 (tannic acid 2; basal diet + 2 g/kg TA) groups on D 42. There were 21 replicates per treatment and core for each parameter was analyzed using the Kruskal–Wallis test followed by the Dwass–Steel–Critchlow–Fligner *post hoc* test.

No differences were observed in average breast muscle myopathy scores for white striping, woody breast, spaghetti meat, or hemorrhagic lesions as shown in Figure 7 (*p* > 0.1). The supplementation of TA linearly reduced pH of the breast meat (*p* < 0.05; Table 11) and linearly increased redness (a*) (*p* < 0.01). The TA2 group had significantly greater redness value compared to the TA0 group. The yellowness (b*) tended to be increased due to the supplementation of TA (*p* = 0.086). The supplementation of TA quadratically modulated cooking loss (*p* < 0.05) in the breast meat, and the TA2 group tended to have lower cooking loss compared to the TA1 group (*p* = 0.054).

**TABLE 11:** The pH, meat color (L*, a*, b*), drip loss (%), thawing loss (%), and cooking loss (%) in the breast meat of broilers fed diets supplemented with tannic acid^a^.

								Polynomial contrast^c^	
Items	TA0	TA0.25	TA0.5	TA1	TA2	SEM	*p*-value^b^	Linear	Quadratic
pH	6.02	6.06	6.02	5.99	5.96	0.08	0.225	0.037	0.944
Lightness (L*)	58.21	57.60	57.64	59.24	57.94	1.6	0.319	0.701	0.319
Redness (a*)	0.616^b^	1.055^a^ ^,^ ^b^	0.755^b^	1.041^a^ ^,^ ^b^	1.765^a^	0.57	0.008	0.001	0.471
Yellowness (b*)	13.53	13.81	13.05	14.27	14.40	1.18	0.220	0.086	0.933
Drip loss	2.72	2.93	2.81	2.48	2.80	0.90	0.911	0.865	0.626
Thawing loss	2.83	2.77	2.72	3.26	2.43	0.51	0.651	0.574	0.292
Cooking loss	29.97^a^ ^,^ ^b^	30.66^a^ ^,^ ^b^	30.85^a^ ^,^ ^b^	32.72^a^	27.99^b^	2.78	0.054	0.168	0.011

^a^ TA0 (tannic acid 0; basal diet without TA); TA0.25 (tannic acid 0.25; basal diet + 0.25 g/kg TA); TA0.5 (tannic acid 0.5; basal diet + 0.5 g/kg TA); TA1 (tannic acid 1; basal diet + 1 g/kg TA); and TA2 (tannic acid 2; basal diet + 2 g/kg TA).

^b^ Treatment groups (21 replicate per treatment) were compared using PROC MIXED, followed by the Tukey’s individual comparison test. Different letters in the same row means significant differences (*p* < 0.05) among the treatments.

^c^ Orthogonal polynomial contrasts were conducted to assess the significance of linear or quadratic effects of the supplementation of TA in broilers.

## 4 Discussion

Our previous study showed that higher than 1 g/kg TA exhibited antinutritional effects to reduce growth performance, whereas 0.5 g/kg TA increased antioxidant capacity in broilers on D 21 (Choi et al., 2022d). The supplementation of TA (0.5 g/kg–2.75 g/kg) improved gut barrier integrity and decreased oocyst shedding in broilers infected with *Eimeria maxima* (Choi et al., 2022c). The supplementation of TA (1 g/kg–2 g/kg) enhanced growth performance and gut health *via* antimicrobial and immunostimulatory effects in broilers infected with *Salmonella* Typhimurium (Choi et al., 2022a). Based on our previous studies, we aimed to evaluate the efficacy of the supplementation of TA in broilers raised for 42 days in floor pens in the current study to simulate actual conditions of broiler production. Sampling points were determined at D 18 and D 36 to represent starter/grower phase and finisher phase, respectively, in the current study. Therefore, the purpose of the study was to investigate the effects of the TA supplementation (up to 2 g/kg TA) on growth performance, intestinal morphology, activities of brush border digestive enzymes, AID of nutrients, relative mRNA expression of tight junction proteins and nutrient transporters, liver antioxidant capacity, bone health, body composition, and meat yield and quality in broilers on D 42.

In the current study, diets were crumbled and pelleted in the starter and grower/finisher phases, respectively. Almost all commercial broiler feeds are crumbled or pelleted in current broiler production (Brickett et al., 2007). The pelleting processing includes steaming (e.g., conditioning) with high temperature and pelleting (e.g., agglomeration) to produce large particles from small particles (Abdollahi et al., 2013). In these harsh conditions, stability and molecular or physical traits of feed additives can be altered (Choi et al., 2020a). Kim et al. (2010) reported that thermal process (e.g., autoclave heat) improved antioxidant capacity and antimicrobial effects of TA, and our unpublished data showed that pelleting temperature (80°C) improved antimicrobial effects of TA against *S.* Typhimurium in *vitro* conditions. However, steaming and agglomeration during feed processing may induce interactions of TA and nutrients (e.g., proteins, polysaccharides, etc.), which can decrease nutrient utilization in the gastrointestinal tract of chickens.

In the current study, supplementation of TA linearly reduced feed intake of broiler chickens in the starter phase while supplementation of TA did not influence feed intake in the grower and finisher phases. Tannins are known to induce astringent taste by forming complexes with salivary proline-rich proteins, which can decrease feed palatability and feed intake in animals (Treviño et al., 1992; Lee et al., 2010). In the starter and grower phases, BW and feed efficiency were linearly reduced, but no statistical differences were observed in the finisher phase in broilers fed dietary TA in the current study. These results is in consistent with our previous study suggested that young broilers are less tolerant to the intake of TA (Choi et al., 2022b). These results had different trends from our previous studies as follows. Choi et al. (2022d) reported that higher than 1 g/kg TA started to linearly reduce BW of broilers on D 21. However, growth retardation effects of TA were exhibited from 0.25 g/kg TA in the current study. Potentially, the pelleting process may have induced the interaction between TA and dietary nutrients (e.g., proteins). Although AID was not measured on D 18 in the current study, nutrient digestibility would have been severely decreased by the supplementation of TA. However, the supplementation of TA improved AID of DM, OM, ash, and CP on D 36 potentially as compensation effects and did not alter growth performance in the finisher phase of the current study, indicating that older birds have more tolerance to the TA supplementation. Potentially, mature gastrointestinal tracts (e.g., lower pH and higher pancreatic enzymes) may have hydrolyzed TA-nutrient complex in broilers on D 36 (Adamczyk et al., 2011). In contrast, a previous study by Tonda et al. (2018) reported that the supplementation of TA extract improved BWG and feed efficiency in cocci-vaccinated (live vaccine) broilers fed pelleted feed on D 0 to 21. This would be because live vaccines are known to spread coccidiosis in a flock and can decrease growth performance, which can provide challenging conditions to chickens, and this indicates the supplementation of TA extract could be effective in challenging conditions (Greif, 2000). However, the basal diets included a coccidiostat (monensin sodium; 500 mg/kg) to exclude the anti-coccidial effects of TA and was conducted in a hygienic laboratory scale facility, and therefore there would be limited gap to improve growth performance of broilers, which potentially explains reduced or maintained growth performance in broilers supplemented with TA in the current study.

On D 18, the TA supplementation linearly increased jejunal VH:CD and quadratically decreased ileal VH:CD along with reduced CD in the current study. Increased VH:CD indicates augmented nutrient digestion and absorption in chickens (Abd El-Hack et al., 2020). However, if increased VH:CD was accompanied with decreased CD, it cannot be considered as beneficial effects because deeper CD suggests more proliferation and differentiation of stems cells, which would move to the tip of the villus (Liu et al., 2020). Potentially, the TA supplementation caused an impairment in the development of intestinal morphology by decreasing nutrient utilization *via* forming complex with nutrients (e.g., proteins) (Shinde et al., 2015).

Activities of sucrase, a brush border digestive enzyme, in the jejunum tissue were quadratically increased by the TA supplementation on D 18 in the current study, which suggests that appropriate dosages of TA can still improve gut development in broilers (Yang et al., 2008). Moreover, relative mRNA expression of *MUC2* and nutrients transporters including *B0AT1*, *SGLT1*, *PepT1*, and *EAAT3* were linearly and quadratically increased by the TA supplementation. These data indicate that nutrient utilization capacity of the jejunum could be enhanced by the TA supplementation, but limited availability of nutrients due to interactions between TA and nutrients would be the main factor to decrease growth performance of broilers in the starter and grower phases. Otherwise, reduced availability of nutrients for intestinal absorption due to the formation of TA-nutrient complexes in the luminal side may have increased mRNA expression of nutrients as a resistant reaction to increase nutrient absorption in the gastrointestinal tract (Pinheiro et al., 2013).

Tight junction proteins and *MUC2* are closely associated with gut barrier integrity of broilers (Choi et al., 2020b). In the present study, the TA supplementation linearly and quadratically increased relative mRNA expression of genes related to gut barrier integrity including *ZO2*, *CLDN2*, *JAM2*, and *MUC2* in the jejunum. According to our previous study, the TA supplementation decreased gut permeability in broilers infected with *E. maxima* (Choi et al., 2022c). A previous study by Yu et al. (2020) reported that the supplementation of TA improved gut barrier integrity in weaned piglets. These results suggest that the supplementation of TA has potential to increase gut barrier integrity in broilers.

There were no differences in TAC, concentrations of GSH and GSSG, activities of SOD in the liver on D 18 and 36 among the treatments in the present study. Many *in vitro* studies showed that TA, a polyphonic compound, has strong antioxidant capacity (Andrade Jr et al., 2005; Gülçin et al., 2010). However, direct antioxidant effects of TA in the chickens were in question. This is because TA should stay inside of the chicken body for a sufficient time by maintaining appropriate forms to exhibit antioxidant capacity (Karakaya, 2004). Deposition of TA in the internal organs (e.g., liver) in broiler chickens should be further investigated. Choi et al. (2022d) showed that the supplementation of TA at 0.5 g/kg indirectly improved antioxidant system by enhancing activities of SOD in the liver. The differences would be originated from the pelleting process, which may reduce bioavailability of TA by forming TA-nutrient complexes. However, under the heat stress condition, the supplementation of TA (10 g/kg) showed potential to improve antioxidant capacity in broilers (Ebrahim et al., 2015).

In the present study, the supplementation of TA linearly decreased alpha diversity indices including faith’s phylogenetic diversity (communities’ evolutionary distance; D 18 and 36), observed features (richness; D 18 and 36), pielou evenness (evenness; D 36), and shannon entropy (richness and evenness; D 36). While it is still controversial, lower alpha diversity may indicate less stable and unmature microbial communities in the gastrointestinal tract of animals (Ebrahim et al., 2015). Moreover, beta diversity indices (unweighted and weighted unifrac) showed that different dosages of TA could modulate cecal microbial communities in broilers.

The relative abundance of the phylum Actinobacteria was linearly increased by the supplementation of TA on D 18 in the current study. The phylum Actinobacteria includes *Bifidobacteria* spp., which can improve gut barrier integrity and immune system of animals and is considered as a beneficial phylum in animals (Binda et al., 2018). On D 18, the supplementation of TA linearly reduced relative abundance of the family Enterobacteriaceae, which includes diverse pathogens such as *Salmonella* spp., *Shigella*, *Escherichia coli*, etc. Consistently, our previous study reported that the TA supplementation reduced cecal *Salmonella* Typhimurium load in the starter phase of broilers (Choi et al., 2022a). Moreover, the TA supplementation quadratically increased the relative abundance of the families Lachnospiraceae and Ruminococcaceae, which have an important role in maintain gut homeostasis by producing volatile fatty acid *via* fiber degradation (Biddle et al., 2013). Consistently, Koo and Nyachoti (2019) reported that the TA supplementation enhanced cecal volatile fatty acid production in pigs. However, on D 36, a ratio of the phyla Firmicutes and Bacteroidetes was quadratically reduced by the supplementation of TA in the current study. The lower ratio of the phyla Firmicutes and Bacteroidetes suggests a lower capacity of fiber degradation and production of short chain fatty acids, important energy sources for the host animals (Singh et al., 2012). In the current study, the TA supplementation linearly decreased the relative abundance of the families Christesenellaceae and Erysipelotrichaceae, which have an important role in fiber degradation to produce short chain fatty acids (Wasti et al., 2021). However, the TA2 group significantly increased the relative abundance of the family Lachnospiraceae compared to the TA0.25 and TA0.5 groups, and the TA supplementation linearly increased the relative abundance of the family Bacillaceae, which are positively correlated with growth performance and feed efficiency (Moula et al., 2018). While the supplementation of TA reduced the relative abundance of the families Christensenellaceae and Erysipelotrichaceae, the TA supplementation still increased the relative abundance of the families Lachnospiraceae and Bacillaceae in the current study.

Ammonia (NH_3_) in poultry houses can negatively affect the health of chickens and humans as well as harm the environment (Naseem and King, 2018). Chickens synthesize uric acid as the end product of purine and protein metabolism, and uric acid is converted into ammonia *via* microbial fermentation in the ceca or in the litter (Kim and Patterson, 2003a; b; Naseem and King, 2018). In the current study, we hypothesized that litter ammonia concentration could be reduced by the supplementation of TA because the TA supplementation increased AID of CP on D 36. Crude protein digestibility is closely associated with litter ammonia concentration (Brink et al., 2022). Moreover, Arzola-Alavarez et al. (2020) reported that the addition of pine bark tannin in the litter reduced ammonia accumulation in the poultry litter. Unabsorbed TA could be excreted to the litter and potentially modulate ammonia concentration in the litter. However, no differences were observed in the litter ammonia on D 42. The TA1 group had a significantly higher FPD score compared to the TA2 group, which implies that the supplementation of TA can modulate FPD score in broilers. Litter ammonia and FPD are closely associated (Youssef et al., 2011), and our current study also showed that litter ammonia and FPD score had similar trends. Otherwise, BW could simply affect litter ammonia because bigger birds would excrete more manure in the litter. The TA1 group had numerically close BW compared to the TA0 group, whereas the TA2 group had the numerically lowest BW on D 42 in the current study. These results may explain the trends of litter ammonia and FPD score of broilers fed diets supplemented with TA in the current study.

Our previous study showed that there was only a linear tendency (0.05 < *p* ≤ 0.10) to reduce bone health parameters including BMD and BMC in broilers on D 21, but no statistical differences were observed between the groups fed 0 g/kg TA and 2.5 g/kg TA (Choi et al., 2022d). However, in the present study, BMD and BMC were linearly reduced by the supplementation of TA in broilers, and 2 g/kg TA supplementation significantly reduced BMD and BMC compared to the control group on D 42. Possibly, the TA supplementation might have reduced utilization of calcium, phosphorous, and iron, which are important minerals for bone formation in broilers (Hassan et al., 2003; Afsana et al., 2004; Katsumata et al., 2009; Shang et al., 2015). Moreover, the pelleting process may have induced more formation of TA-mineral complexes, which dramatically reduced BMD and BMC in broilers. A previous study by Tomaszewska et al. (2018) also reported that inclusion of low-tannin faba bean (condensed tannins) negatively affected tibia traits (weight, reduction of the cross section area, and wall thickness) in broilers.

In the present study, the fat percentage measured by DEXA was linearly increased and the ratio of lean to fat decreased in broilers on D 42. This result is in stark contrast to our previous results reporting that the supplementation of TA increased the ratio of lean to fat in broilers on D 21 (Choi et al., 2022d). Discrepancies between the findings of these studies could have originated from differences in the supplementation period and age of birds (D 42 vs. D 21) and feed form (pelleted vs. mash). In our previous study (Choi et al., 2022d), fat accumulation was reduced in broilers D 21 due to less production of cecal volatile fatty acids, which potentially resulted in an imbalance of energy homeostasis. However, in the current study, the pelleting process may have decreased nutrient utilization by inducing the formation of TA-nutrient complexes, and young broilers, which did not have mature enough gastrointestinal tract to hydrolyze TA-nutrient complexes, may have decreased digestibility of energy and nutrients. To compensate limited growth rate at young stage due to reduced nutrient and energy utilization by the supplementation of TA, the broilers may have altered their body metabolism to increase the accumulation of fat (Kobylińska et al., 2022). Consistently, a previous study by Starčević et al. (2015) also showed that the supplementation of TA (5 g/kg) increased fat accumulation in the breast and thigh meat in broilers on D 35.

In the current study, absolute and relative weight of abdominal fat were increased by the supplementation of TA, which is consistent with DEXA results. The abdominal fat weight is a dependable parameter to represent body fat content because abdominal fat is the main and largest area (up to 4% of BW) of fat accumulation in broilers (Thomas et al., 1983; Fouad and El-Senousey, 2014). Furthermore, the supplementation of TA resulted in linearly increased leg meat and linearly decreased breast meat yield (*p* = 0.168), while statistical differences were not observed. Leg meat had higher fat content compared to breast meat (Pikul et al., 1985). Potentially, increased fat metabolism in broiler body by the supplementation of TA resulted in increased leg meat yield and decreased breast meat yield to increase fat accumulation in chickens. Fatty broiler meat and low yield of breast meat are not preferred in modern broiler production (Fouad and El-Senousey, 2014). Low pH of the breast meat results in higher lightness, lower redness, and higher yellowness by decreasing water binding capacity (Allen et al., 1997; Qiao et al., 2001). Our current study showed that the supplementation of TA decreased pH, increased redness and yellowness, and reduced cooking loss in the breast meat. This would be due to immatureness of breast meat and low growth performance caused by the TA supplementation. According to Bianchi et al. (2007), smaller broilers had lower pH and higher redness when compared to the bigger broilers. However, the lightness, an important factor to indicate pale, soft, and exudative (PSE)-like condition in poultry meat (Petracci et al., 2004), was not modulated due to the supplementation of TA in the current study. Moreover, the supplementation of TA did not dramatically altered those meat quality parameters, and the values were in still normal range (Hertanto et al., 2018). No differences were observed in the breast muscle myopathies such as white striping, woody breast, spaghetti meat, and hemorrhagic lesion in the current study. However, numerical reductions in the scores of spaghetti meat and hemorrhagic lesion in the breast meat would be associated with retarded growth rate and immatureness of breast meat in broilers fed dietary TA because breast meat myopathies are frequently observed in the fast-growing broilers (Caldas-Cueva and Owens, 2020). Therefore, the supplementation of TA did not significantly alter meat quality of broiler chickens.

In the current study, discrepant results from our previous studies and negative effects of the TA supplementation would be mainly attributed to the pelleting process on diets, which induced the formation of TA-nutrient complexes. In order to minimize or inhibit the interaction of TA and dietary nutrients during pelleting process, the encapsulation of TA can be a potential strategy (Choi et al., 2020a). Encapsulation process can provide protection for TA and release TA in the target site of the gastrointestinal tract, where many pathogens propagate (e.g., lower gut) (Choi et al., 2020a). A previous study by Wang M. et al. (2020) reported that encapsulated TA showed beneficial effects on gut health and microbiota of weaned piglets. Future studies should include: 1) investigation of appropriate methods to encapsulate TA and its stability during pelleting process and in the gastrointestinal tract; and 2) investigation into the effects of encapsulated TA on growth performance and gut health in broilers on D 42.

## 5 Conclusion

The TA supplementation up to 2 g/kg in pelleted diets positively affected gut microbiota, enhanced brush border digestive enzyme activities, upregulated genes related to gut barrier integrity and nutrient transportation in the starter/grower phases, and improved nutrient digestibility in the finisher phase. However, the supplementation of TA decreased overall growth performance and feed efficiency, increased fat accumulation, and negatively affected gut microbiota, bone health, and meat production in broilers on D 42. Therefore, further processing should be applied on TA to enhance their potential beneficial effects on broilers.

## Data Availability

The authors acknowledge that the data presented in this study must be deposited and made publicly available in an acceptable repository, prior to publication. Frontiers cannot accept a manuscript that does not adhere to our open data policies.
